# DNA Mutations Mediate Microevolution between Host-Adapted Forms of the Pathogenic Fungus *Cryptococcus neoformans*

**DOI:** 10.1371/journal.ppat.1002936

**Published:** 2012-10-04

**Authors:** Denise A. Magditch, Tong-Bao Liu, Chaoyang Xue, Alexander Idnurm

**Affiliations:** 1 Division of Cell Biology and Biophysics, School of Biological Sciences, University of Missouri-Kansas City, Kansas City, Missouri, United States of America; 2 Public Health Research Institute Center, University of Medicine and Dentistry of New Jersey, Newark, New Jersey, United States of America; University of Birmingham, United Kingdom

## Abstract

The disease cryptococcosis, caused by the fungus *Cryptococcus neoformans*, is acquired directly from environmental exposure rather than transmitted person-to-person. One explanation for the pathogenicity of this species is that interactions with environmental predators select for virulence. However, co-incubation of *C. neoformans* with amoeba can cause a “switch” from the normal yeast morphology to a pseudohyphal form, enabling fungi to survive exposure to amoeba, yet conversely reducing virulence in mammalian models of cryptococcosis. Like other human pathogenic fungi, *C. neoformans* is capable of microevolutionary changes that influence the biology of the organism and outcome of the host-pathogen interaction. A yeast-pseudohyphal phenotypic switch also happens under *in vitro* conditions. Here, we demonstrate that this morphological switch, rather than being under epigenetic control, is controlled by DNA mutation since all pseudohyphal strains bear mutations within genes encoding components of the RAM pathway. High rates of isolation of pseudohyphal strains can be explained by the physical size of RAM pathway genes and a hypermutator phenotype of the strain used in phenotypic switching studies. Reversion to wild type yeast morphology *in vitro* or within a mammalian host can occur through different mechanisms, with one being counter-acting mutations. Infection of mice with RAM mutants reveals several outcomes: clearance of the infection, asymptomatic maintenance of the strains, or reversion to wild type forms and progression of disease. These findings demonstrate a key role of mutation events in microevolution to modulate the ability of a fungal pathogen to cause disease.

## Author Summary

Many diseases are contracted from the environment, rather than from sick people. It is unclear why those species are able to cause disease, since the selective pressures in the environment are presumed to be very different from those found within the host. *Cryptococcus neoformans* is a fungus that causes life-threatening lung and central nervous system disease in approximately one million people each year. The fungus is inhaled from environmental sources. One hypothesis to account for *C. neoformans* virulence is that amoeba are predators for this fungus, and surviving strains are pre-selected to be virulent in the human host. On the other hand, experiments have found that amoeba eat *C. neoformans*. A pseudohyphal cell type can survive, and while protecting against amoeba these cells are unable to cause disease in mouse models. We predicted that the pseudohyphal morphology reflected a change in function of a pathway of genes, and found that all pseudohyphal isolates contain mutations within genes for this pathway. The pseudohyphal trait is unstable, with reversion to normal yeast growth by counter-acting mutations. These mutations can occur during the course of mammalian infection. Our results show that mutation events account for a microevolution system currently described as phenotypic switching, and that mutations, at least under experimental conditions, can regulate pathogen adaptation and influence its host range.

## Introduction

Pathogens across all major microbial groups – viruses, bacteria, fungi and protists – have representative species that owe their success to rapid change during infection or within a population. Microevolution is thus essential to pathogenesis, yet due to its stochastic nature it can be difficult to study and the underlying mechanisms challenging to elucidate.

*Cryptococcus neoformans* is a fungal pathogen that is acquired directly from the environment through inhalation of desiccated yeast cells or the sexual basidiospores. The fungus is found world wide and it causes disease predominantly in immunocompromised individuals, especially AIDS patients [1], [2]. The global mortality rate is estimated at 624,000 per annum [3]. The closely-related species *C. gattii* causes disease mostly in healthy individuals, and is responsible for an ongoing and expanding outbreak of cryptococcosis in the Pacific Northwest of Canada and the United States [4], [5], [6]. Both *Cryptococcus* species are extensively studied, have a suite of experimental resources, and serve as general models for understanding pathogenesis and its evolution in pathogenic eukaryotes [7], [8]. *Cryptococcus* species undergo microevolution *in vitro*, within animal models, and during the course of disease in humans [9], [10], [11], [12].

A current hypothesis is that the *Cryptococcus* species are pre-selected for virulence within mammalian animals because of interactions with predatory microbes like amoeba or nematodes [13], [14], [15], [16]. Evidence for this comes from studies on interactions with non-mammalian hosts. *C. neoformans* has been co-isolated with three different *Acanthamoeba* species [15], [17], [18] and these amoebae can take up the fungus by phagocytosis [19], [20]. Genes that are essential for mammalian virulence are also required for virulence in non-mammalian models [21]. Furthermore, screens of insertional mutants of the fungus with the nematode *Caenorhabditis elegans* identified fungal genes required for nematode viability: deletion of these genes also reduces virulence in mouse models of cryptococcosis [22], [23]. Additional support for the hypothesis comes from passage of *C. neoformans* through a slime mold host, *Dictyostelium discoideum*, since this produces strains with increased virulence in mice [24]. However, one caveat is that *Acanthamoeba* species ingest and kill *C. neoformans*. Surviving subpopulations can be isolated, including one common class that has pseudohyphal cells rather than the normal yeast shape [17]. The pseudohyphal strains were avirulent in animal models [17], [25], [26].

Strikingly, the pseudohyphal phenotype is not always stable. One of the eight pseudohyphal strains originally isolated, after wild type strains were exposed to amoeba, was inoculated into mice and it demonstrated wild type virulence [17]. Closer examination revealed, to quote directly, that “a high percentage of the cells in the inoculum of this isolate had reverted to the encapsulated yeast form” [17], a description of an unstable trait governed by epigenetics or microevolution. In repeat experiments, including use of different wild type strains of *C. neoformans* and a different species of *Acanthamoeba*, pseudohyphal isolates were again obtained: the phenotype exhibited instability in some, but not all, strain backgrounds [15], [25]. More recently, a similar pseudohyphal morphology was reported from *in vitro* experiments, and that it too could revert back to wild type at a high frequency (e.g. an average of 1 revertant per 1600 colonies) [27]. This high frequency of phenotypic change is referred to as phenotypic switching. The basis for this rapid evolution in *C. neoformans* remained unknown.

The pseudohyphal morphology of strains from amoeba or phenotypic switching appear similar to those of *C. neoformans* strains with loss-of-function mutations in the RAM/MOR pathway of genes [28]. This pathway is conserved in eukaryotes and characterized primarily in *Saccharomyces cerevisiae* where the abbreviation is from Regulation of Ace2p activity and cellular Morphogenesis [29], [30]. In *Schizosaccharomyces pombe* the pathway is known as MOR, for the Morphogenesis-related NDR kinase network. The pathway comprises six components, centered around the action of two protein kinases Cbk1 and Kic1 and the accessory proteins Sog2, Hym1 and Mob2. The large size and physical interaction of Tao3 with Cbk1 and Kic1 suggests that Tao3 may act as a scaffold protein [29], [31]. Within the fungi, the pathway can have dramatically different effects on cell type, for instance promoting cell polarity in the ascomycete yeast *S. cerevisiae* whereas inhibiting it in the basidiomycete yeast *C. neoformans* [28], [29]. It is required for virulence in plant and human pathogenic fungi [32], [33], [34], [35], and polymorphisms in *KIC1* recently emerged from genome wide association studies between clinical and non-clinical isolates of *S. cerevisiae* [36]. The role of the RAM pathway in pathogenesis has been most thoroughly analyzed in *Candida albicans*, in which components mediate cell separation and polarity, and thus mutations block filamentation, impair biofilm formation and surface adhesion, and reduce virulence [34], [37], [38], [39], [40], [41], [42]. Mutation of the RAM pathway in *C. neoformans* causes a pseudohyphal morphology and other phenotypic changes [28], although effects on virulence had not been tested.

We hypothesized that the RAM pathway is affected in strains that “switch” morphology either *in vitro* or upon exposure to amoeba, and that the analysis of this process would provide insight into the mechanism of microevolution. In this study, we identify mutations within a RAM pathway gene in original pseudohyphal mutants derived from amoeba and phenotypic switching. We recapitulate the isolation of pseudohyphal strains by both means, and find that these strains all have mutations in the RAM pathway. A strain derived from amoeba with a point mutation in the *MOB2* gene was unable to cause disease in a murine model, unless during the course of infection the mutation reverted to wild type. Switching back to yeast morphology relies on a multifactor system for microevolution that is also driven by DNA mutations. These findings demonstrate that DNA mutation contributes to fungal pathogenesis.

## Results

### Pseudohyphal isolates of *C. neoformans* of different origins have similar phenotypes

**Figure 1:**
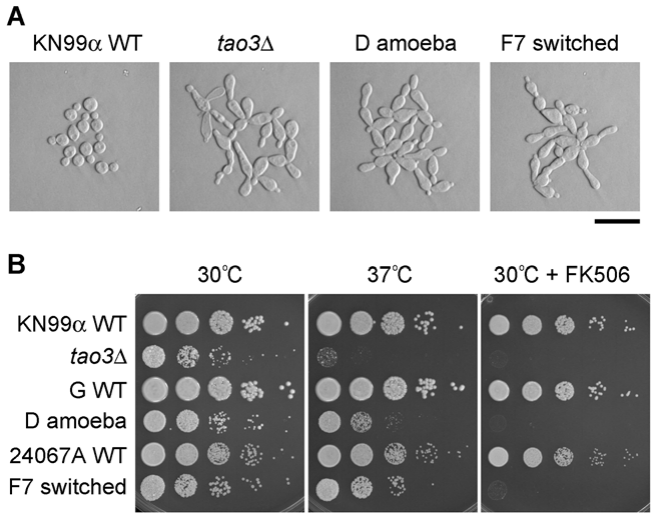
Pseudohyphal strains derived from three origins share similar characteristics. The *tao3*Δ strain is in the KN99α background. Strains C, D and E were isolated after exposure to amoeba, probably in the strain G background. All three strains derived from exposure to amoeba exhibited identical phenotypes, so just D is illustrated. F7 is a phenotypic switching isolate in the ATCC 24067A background. WT = wild type. **A.** Light microscopy of cells (bar = 50 µm). **B.** Growth under different conditions. Cells were 10-fold serially diluted and spotted onto YPD medium with or without FK506 (1 µg/ml), and grown for two days at 30°C or 37°C.

The *C. neoformans* species consists of two varieties, var. *grubii* (serotype A) and var. *neoformans* (serotype D). Pseudohyphal strains have been isolated from both varieties (table S1). Three isolates of *C. neoformans* var. *grubii* from the 1970s that originated after exposure to amoeba (strains C, D and E; ATCC 42343–42345 [17]) and one *C. neoformans* var. *neoformans* isolate from the 1990s that originated from a phenotypic switching study (strain F7 [27]) were compared to defined deletion strains of the RAM pathway genes. The strains had similar pseudohyphal cell morphologies distinguishing them from wild type yeast cells (Fig. 1A). Most strains showed decreased growth at mammalian body temperature (Fig. 1B). Consistently, all were highly sensitive to the immunosuppressive chemical FK506 (Fig. 1B). This drug inhibits the calcineurin pathway, the impairment of which is synergistically lethal with RAM pathway mutation in *C. neoformans* [28]. The similarity in phenotypes between the strains isolated from amoeba and phenotypic switching with defined deletion strains suggests the same genes or pathways are affected, and that this could be the RAM pathway.

### Pseudohyphal isolates derived from amoeba or phenotypic switching have premature stop codons in the RAM pathway *TAO3* gene

We hypothesized that the historical pseudohyphal strains represent loss of the RAM pathway. The nature of the deficiency was sought in the strains that had been isolated from amoeba and the phenotypic switched isolate. Epigenetic or microevolutionary changes can arise from a suite of different causes. As a first approach towards gene identification, constructs containing wild type copies of four of the genes in the pathway (*MOB2*, *CBK1*, *KIC1* and *SOG2*) were introduced into strains C, D, E and F7 with the endeavor to identify the affected gene by expressing additional copies. None of the four genes restored growth to the wild type yeast form, whereas these constructs complemented deletion strains. A fifth RAM pathway gene, *HYM1*, has not emerged as a RAM component in *C. neoformans* from random mutant screens, and has thus far eluded gene replacement experiments. The sixth component of the pathway, *TAO3*, is large and it was a challenge to generate a vector for this gene for transformation experiments. However, the lack of evidence for a direct role by the other members of the RAM pathway provoked closer examination of the *TAO3* gene in the pseudohyphal strains.

**Figure 2:**
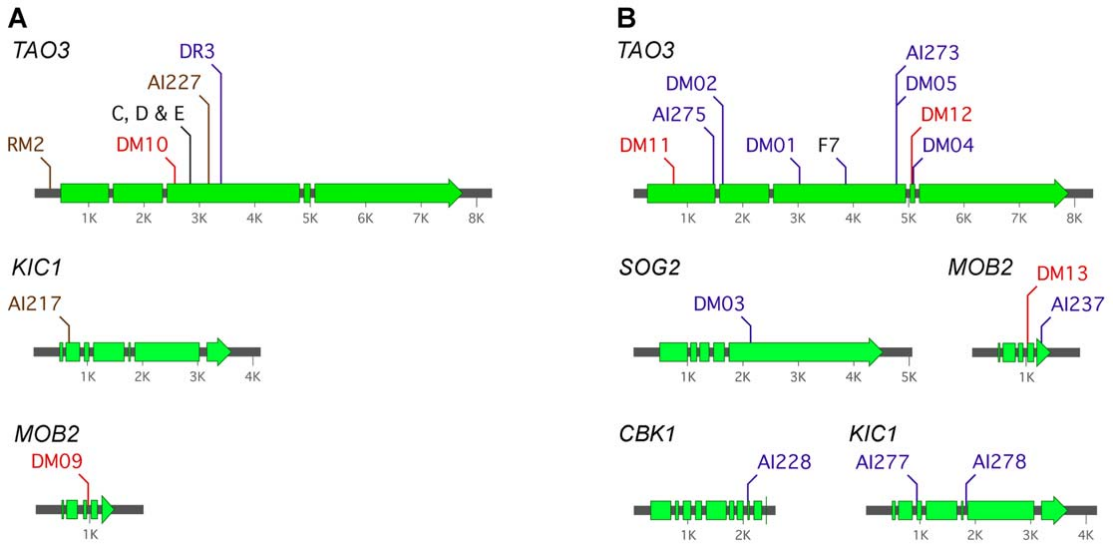
Pseudohyphal strains of *C. neoformans* derived from independent sources bear RAM pathway mutations. The *TAO3* gene is most commonly mutated in pseudohyphal strains. Mutations in “historical” isolates are in black, spontaneous mutants in blue, T-DNA insertional mutants in brown, and from amoeba in red. **A.** *C. neoformans* var. *grubii*, in the strain G or KN99α backgrounds. The strains C, D and E were isolated from exposure to amoeba in the 1970s. **B.** *C. neoformans* var. *neoformans*, all in the ATCC 24067A strain background. Strain F7 is from a phenotypic switching study [27]. Sequence information for these mutations is provided in dataset S1.

The *TAO3* gene was sequenced from the four historical pseudohyphal strains. The three strains isolated after exposure to amoeba (strains C, D and E) all contained an identical predicted g-t base pair substitution, that results in a codon for glutamic acid being substituted for a premature stop codon (Fig. 2A; dataset S1). The wild type strain for the three amoeba derived isolates is unknown, but is likely to be strain G (ATCC 42437) based on co-deposition of this isolate with C, D and E to the American Type Culture Collection. The *TAO3* gene was sequenced from strain G to confirm that the stop codon is not present. The *TAO3* gene from the strains C, D, E and G strains, with the exception of the stop codon in three isolates, was identical in sequence to the gene from *C.n.* var. *grubii* strain H99 sequenced by the Broad Institute (CNAG_03622). Sequence analysis of strain F7, the phenotypic switched strain, has an allele of *TAO3* bearing a predicted a-t bp substitution that also causes a premature stop codon (Lys-Stop, Fig. 2B and dataset S1). The wild type parent for F7 is strain ATCC 24067A, and *TAO3* was amplified and sequenced from this strain. The wild type strain does not contain the stop codon. Thus, the historical pseudohyphal strains derived from exposure to amoeba or phenotypic switching are *tao3* mutants.

### Reconstitution of *TAO3* by replacing the stop codon restores wild type yeast growth

**Figure 3:**
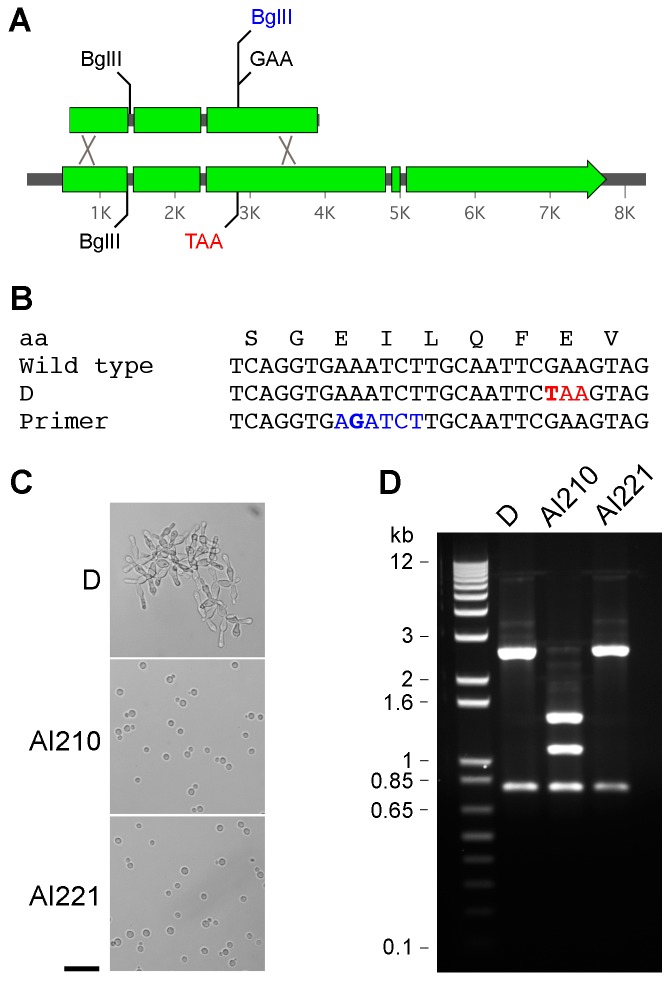
A *tao3* point mutation can be reconstituted by homologous recombination with a wild type fragment of *TAO3*. Strain D was transformed with a modified fragment of *TAO3* by biolistic bombardment. Strains were plated on FK506 to select for wild type growth. **A.** and **B.** To ensure gene replacement rather than a reversion event, a BglII site (grey, a-g mutation in bold) was engineered near the affected codon. **C.** Morphology of strain D and two FK506^R^ strains (bar = 50 µm). **D.** Amplification by PCR and restriction digestions with BglII. Size markers = Invitrogen 1 kb+ ladder. Strain AI210 has undergone homologous recombination, while strain AI221 is a spontaneous revertant.

While the stop codons within the *TAO3* gene are consistent with impaired RAM function, we aimed to demonstrate that these mutations cause the pseudohyphal phenotype. A Mendelian genetic segregation approach was first considered. However, strain F7 and the three amoeba-derived strains are infertile and thus genetic linkage tests were not feasible. Next, a construct was generated by overlap PCR that introduces a silent point mutation into the coding region adjacent to the Glu-stop codon, and at the same time introduces a new BglII restriction enzyme site (Fig. 3A, B). The rationale behind this approach was that the chance of two identical nucleotide changes occurring in independent strains is highly unlikely, and this construct could be used to distinguish reconstituted strains from reverted strains. The *TAO3^BglII^* construct was transformed into strain D using a biolistic apparatus that facilitates homologous gene replacement events. After transformation the cells were plated on FK506-containing media. Strains were isolated with the wild type yeast morphology, and when the *TAO3* fragment was amplified and the PCR product digested with BglII, a subset of strains now contained a novel BglII restriction enzyme site (Fig. 3C, D). This indicates successful gene targeting and reconstitution to a wild type copy of the gene, and that the pseudohyphal phenotype is due to mutation of *TAO3*.

### Isolation of new pseudohyphal strains and their characterization reveals *TAO3* as the primary target for mutation, although multiple genes of the RAM pathway can be affected

Additional pseudohyphal strains were sought in order to explore the basis of this trait and whether or not it was specific to the *TAO3* gene. Two *C.n.* var. *grubii* candidate *TAO3* mutant strains isolated in a previous study [28], but not further characterized, were examined through PCR and DNA sequencing (Fig. 2; dataset S1). One has a T-DNA insertion in the promoter of the *TAO3* gene, and the other has a deletion of 7 bp (gcgtagc). Two pseudohyphal T-DNA mutants were isolated during other experiments. One contains an insertion in the *KIC1* gene and the other in *TAO3*. In addition, strains with complete deletion of *TAO3* were generated in three backgrounds (*C.n.* var. *grubii* KN99α and strain G, and *C.n.* var. *neoformans* ATCC 24067A). Overlap PCR products were created to replace the *TAO3* ORF with the nourseothricin acetyltransferase cassette via biolistic transformation and homologous recombination. The *tao3* point mutant strains have the same phenotype as complete deletion or T-DNA insertion alleles, consistent with complete loss-of-function.

We screened and isolated 13 spontaneous pseudohyphal strains in the *C.n.* var. *neoformans* ATCC 24067A strain, six using a UV-induction method [27] and seven by plating colonies for random mutation events (table S1). The strains were transformed with the wild type copies of *CBK1*, *KIC1*, *MOB2* or *SOG2* to test for complementation, and/or the *TAO3* gene from these strains was sequenced. Eight contained changes in the *TAO3* sequence, as illustrated in Fig. 2. Complementation experiments implicated mutations in the *KIC1*, *MOB2*, *SOG2* and *CBK1* genes in the remaining five, and these mutations were identified by sequencing those genes (Fig. 2 and dataset S1). The *SOG2* gene in one had a mutation in which a stretch of 16 bp ( tgcacaacgcaactct ) in the fifth exon was duplicated and inserted adjacent to the original sequence. This insertion would result in a frame shift mutation. The *cbk1* mutant bears an a-g mutation in the 3′ g splicing site of an intron. Likewise, the two *kic1* mutants are both bp substitutions within splice sites. *MOB2* was sequenced from the *mob2* mutant, and has a bp deletion that will cause a translational frameshift. In summarizing, *TAO3* is most often mutated in spontaneous pseudohyphal strains but other genes in the RAM pathway can also be affected.

**Figure 4:**
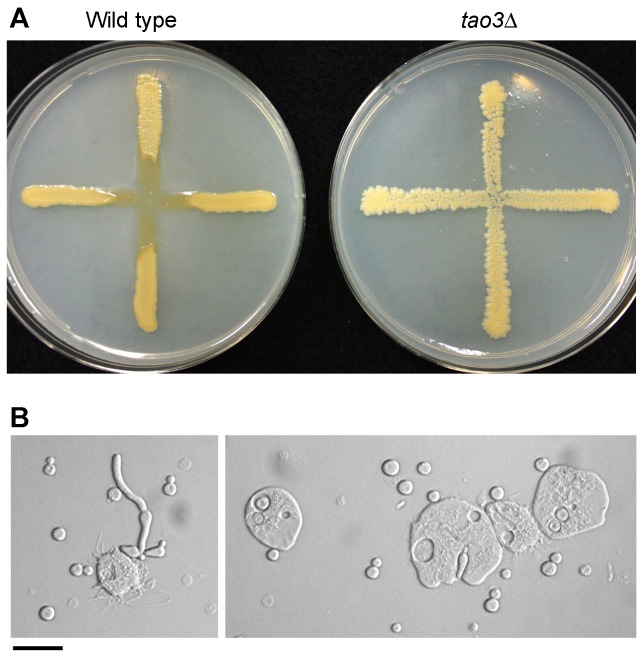
*C. neoformans* RAM pathway mutants are resistant to *Acanthamoeba castellanii*. **A.** *C. neoformans* var. *grubii* wild type (KN99α) and *tao3*Δ deletion (AI235) strains grown on 5% V8 juice agar on 10 cm diameter Petri dishes. Amoeba were dropped at the intersection of the cross and plates incubated for 14 days at room temperature. **B.** Interactions between a mixture of wild type and *tao3* mutant with amoeba, illustrating the size difference between amoeba and pseudohyphal cells (left panel) and internalization of yeast or occasional pseudohyphal cells into amoeba (right panel). Scale bar = 50 µm.

The provenance of the C, D and E strains with pseudohyphal morphology that were isolated in the 1970s by exposing *C. neoformans* to amoeba is not well documented. Isolating RAM mutants using amoeba was tested. The original *Acanthamoeba polyphaga* strain from mouse feces and the *A. palestinensis* strain from pigeon guano, used previously to isolate pseudohyphal colonies, were not saved in a culture collection. The ATCC 30234 strain of *A. castellanii* was co-isolated with *C. neoformans* by Aldo Castellani [18], and is commonly used to study the interactions of amoeba with other pathogenic microbes. First, *C. neoformans* wild type and RAM mutants were exposed to this amoeba in the expectation that a similar interaction would occur as had been described in the 1970s. The outcome of the fungus-amoeba interaction depended on strain background and medium type. In some combinations, such as that illustrated in Fig. 4, the amoeba consumed the wild type strain whereas the RAM pathway mutants were resistant. Second, the wild type strains G (*C.n.* var. *grubii*) and ATCC 24067A (*C.n.* var. *neoformans*) were exposed to the amoeba on proteose peptone agar, and examined 2–3 weeks later for the presence of surviving colonies. A subset was of colonies comprised of pseudohyphal cells. The nature of the mutation within the RAM pathway for those strains was sought through complementation experiments and gene sequencing. Mutations were identified again in the *TAO3* gene, as well as *MOB2* (Fig. 2, dataset S1). These findings thus extend the diversity of the *C. neoformans*/amoeba interaction to include both *C. neoformans* varieties and a third *Acanthamoeba* species. In doing so, the results provide further evidence that the RAM pathway is integral to pseudohyphal morphology, and the isolation of these mutants reflects a common underlying ability across divergent *C. neoformans* strains.

### Multiple mechanisms exist for reversion to wild type morphology in RAM pathway mutants

**Figure 5:**
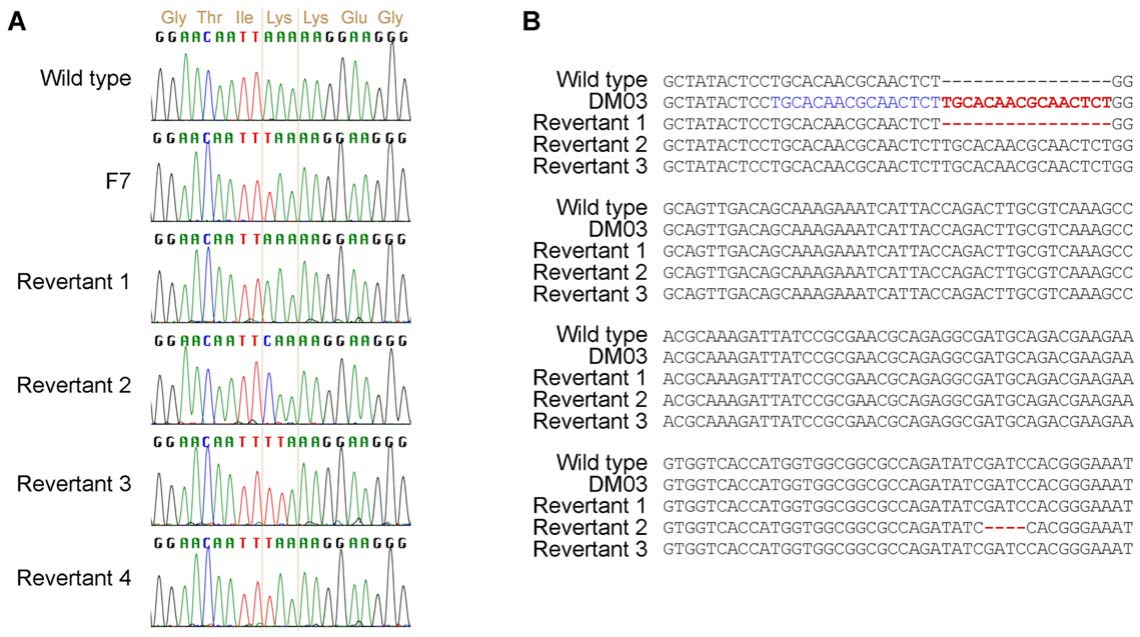
Multiple mechanisms revert mutations in RAM pathway genes to wild type morphology. Mutant strains were plated on FK506 to select for revertants. Regions surrounding the mutations were amplified and sequenced. **A.** Reversion of the stop codon in *tao3* mutant F7. **B.** Reversion of a 16 bp tandem duplication in *SOG2*. The duplicated region is colored blue. Changes between strains that are responsible for the mutation or reversion back to wild type are colored red.

RAM pathway mutants are sensitive to FK506, providing a simple means to select for strains that revert to wild type. Revertants were sought from *tao3* mutant strains D and F7. A high rate of spontaneous resistance to FK506 was observed for strain F7, but not all of these FK506 resistant strains had reverted to the wild type yeast cell morphology. Rather, the strains had acquired FK506 resistance through some other means, possibly through mutation of the gene encoding the FKBP12 protein to which FK506 physically binds [43]. The frequency of reversion and FK506 resistance was noticeably lower in strain D than F7. Part of the *TAO3* gene was sequenced from >30 revertants derived from F7 with the wild type yeast morphology: four types of changes were identified (Fig. 5A). In the first, an a-t bp mutation had restored the original lysine codon. In the second, an a-c bp change replaced the stop codon with a glutamine codon. In the third, the adjacent nucleotide had mutated (a-t) to change the stop codon to a leucine codon. In the fourth, the original stop codon was still present. In addition, another reversion event was also common, leading to a suppression of the RAM phenotype and partial return to the wild type phenotype. These strains were distinctive because of the yellow-colored colonies, with cells exhibiting a lemon-shape and inefficient cell separation. Sequence analysis revealed that the stop codon mutation was still present in *TAO3* in these types of strains. In contrast to the revertants isolated from strain F7, when the region containing the stop codon in *TAO3* was sequenced from 24 revertants of strain D, all 24 still contained the stop codon. Thus, mutation within *TAO3* allows reversion back to wild type, although another mechanism(s) can account for some reversion events.

The pseudohyphal strain DM03 contains a 16 bp duplicated region within the *SOG2* gene, and as such is a different type of mutation compared to the bp substitutions in the *TAO3* gene described above. After selection on FK506 for wild type colonies from strain DM03, three reversion types were observed (Fig. 5B). In one, the duplicated piece of DNA was excised. In a second case, 4 bp were deleted downstream of the insertion event, returning the gene to the correct reading frame and producing an allele encoding 45 different amino acid residues. In the third, the original mutation was still present. Thus, excision of a duplication region or insertions or deletions correcting the open reading frame provide yet additional mechanisms to revert RAM mutants.

Northern blot analysis was use to examine changes in transcript levels or sizes in RAM pathway genes in response to mutation or reversion in a selection of var. *neoformans* and var. *grubii* strains (Fig. S1). The *KIC1* and *TAO3* transcripts were below detectable levels. *CBK1* and *MOB2* have overall constitutive transcript levels. *HYM1* and *SOG2* showed variation in transcript levels, although there was no perfect correlation between loss of a RAM gene and upregulation. Of note, the *mob2* mutant DM09 used in the virulence analysis described below exhibited altered transcript sizes, consistent with a mutation in a predicted intron splice site (Fig. S1).

### The strain used for phenotypic switching studies is a hypermutator

If mutation is the primary source of pseudohyphal strains, it was surprising that they arise at such a high frequency. This together with the observation of high rates of spontaneous resistance to FK506 in the strains in the ATCC 24067A strain background used in phenotypic switching experiments [27], [44], led us to test the mutation rate in this strain. ATCC 24067A is derived by laboratory passage from strain ATCC 24067 [45]. 20 separate cultures of strains ATCC 24067 and ATCC 24067A were plated onto medium to select for spontaneous uracil auxotrophy. The mutation rate for ATCC 24067 was 2.66 per 1×10^8^ (95% confidence interval 1.78–4.75). In contrast, in ATCC 24067A the rate was 67.88 per 1×10^8^ (95% CI 57.06–79.38). Thus, ATCC 24067A has greater than 25 fold higher mutation rate that its progenitor parent ATCC 24067.

**Figure 6:**
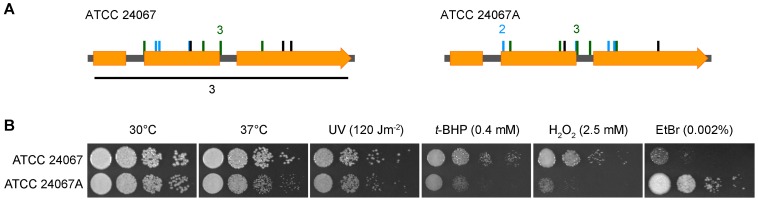
The ATCC 24067A strain used in phenotypic switching is a hypermutator strain. **A.** Profile of *ura5* mutations in strains ATCC 24067 and ATCC 24067A. Orange lines are positions of transversions, green are positions of transitions, and black indicates indels. The black line under ATCC 24067 represents three *ura5* mutants with rearrangements. **B.** Ten-fold serial dilutions of ATCC 24067 and ATCC 24067A plated on different media types, and grown for two days. BHP is *t*-butyl hydroperoxide and EtBr is ethidium bromide.

To ensure that the uracil auxotrophs were due to mutations in the same gene, the *URA5* gene enoding orotate phosphoribosyltransferase was amplified from 15 5-FOA resistant strains derived from separate starting colonies, and sequenced (Fig. 6A; dataset S2). 5-FOA resistance in fungi can result from mutation of either *URA3* or *URA5* homologs; prior studies suggest that *URA5* is the main target in *C. neoformans* [46]. All 30 strains had mutations in *URA5*. Comparing the mutation profiles for ATCC 24067 and ATCC 24067A revealed similarities, e.g. three in each strain background had the same t-c mutation. A main difference was in mutations involving more than one base pair. Two indels for ATCC 24067A were single bp. In contrast, for ATCC 24067 three alleles have large insertions or rearrangements identified by altered or absent PCR products (data not shown). Another three uracil auxotrophs have insertions or deletions between 2 and 18 bp (dataset S2). We interpret this to imply a higher level of bp substitutions in the ATCC 24067A strain. Comparison of the ATCC 24067 and ATCC 24067A strains under stress conditions also revealed altered response to stress agents, especially oxidative stress agents and ethidium bromide (Fig. 6B). We hypothesize that during laboratory passage ATCC 24067A acquired a mutation in a DNA repair pathway gene.

The RAM pathway itself could potentially influence mutation rates, thereby enhancing the rate of reversion. Mutation rates were compared between 15 cultures each of ATCC 24067A and the *tao3* point mutant strain F7. Uracil auxotrophs were isolated at a rate of 42.00 per 1×10^8^ (CI 34.30–53.11) for the ATCC 24067A strain, and 15.74 per 1×10^8^ (CI 13.37–18.93) for the F7 strain. These results suggest that there is no increase in mutation rate in the RAM mutants, since the wild type strain had 2.6 times higher frequency for isolation of uracil mutants compared to the F7 strain. A caveat in this comparison is the challenge of accurate quantification of viable cells for RAM pathway mutants.

**Figure 7:**
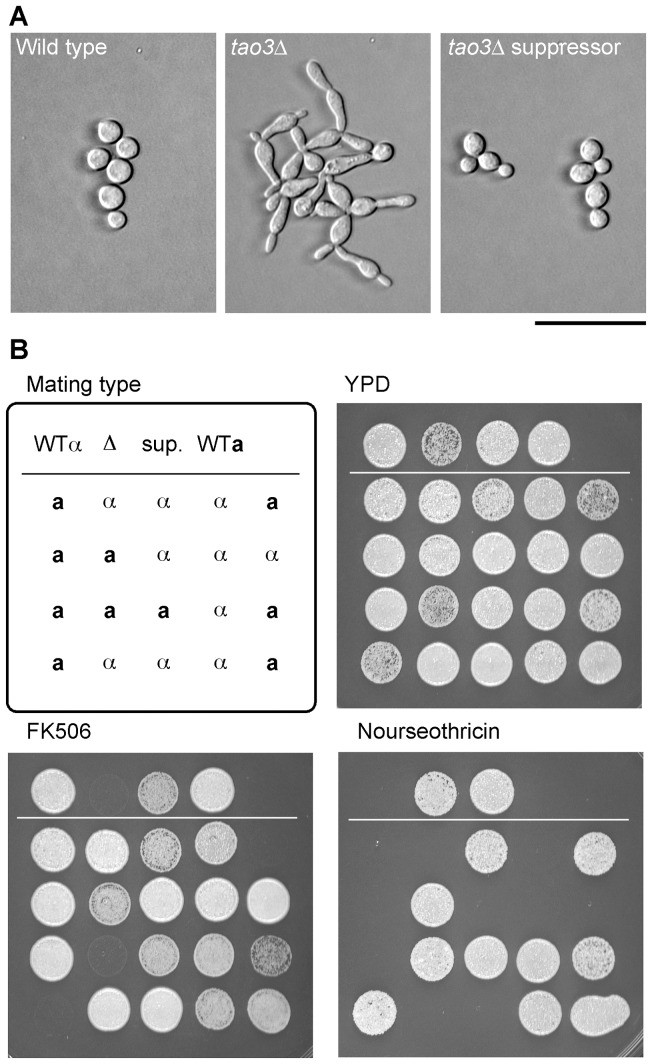
A RAM suppressor phenotype is meiotically stable, and segregates in a Mendelian manner. **A.** Phenotypes of wild type, *tao3::NAT* deletion strain, and suppressor (sup.). Cells in the suppressor strain are often lemon-shaped and have cell separation defects. Bar = 50 µm. **B.** Growth of parental strains and 20 progeny from a cross between suppressor (strain AI235ya) and wild type strain KN99**a**. Mating type was tested by crossing to **a** and α strains as an independent genetic locus segregating in the progeny. Cells were plated onto YPD medium, supplemented with nourseothricin or FK506.

Additional evidence was sought that pseudohyphal strains and reversions are due to mutation events. A “yellow” suppressor or partially-reverted strain was examined by Mendelian genetic analysis. The strain was isolated in the *tao3::NAT* deletion strain background (strain AI235) by selection on FK506. The AI235ya strain was crossed to a wild type of opposite mating type. 20 progeny were obtained: three pseudohyphal NAT^R^ FK506^S^, ten wild type yeast NAT^S^ FK506^R^, and seven “yellow” NAT^R^ FK506^R^ (Fig. 7). These results show that the suppression phenotype is meiotically stable, and the progeny ratio is consistent with its segregation as a single genetic locus. This finding further illustrates that mutation leads to some types of reversion events that are yet to be defined.

### Mutation rates within the RAM pathway genes are similar to those in *URA5*

An alternative hypothesis for the high frequency of isolation of RAM mutants would be if the pathway or parts of it were hot spots for mutations. To test this, aliquots from the identical 20 cultures of strain ATCC 24067A used to isolate uracil auxotrophs were plated onto YPD. Six RAM mutants were identified from ∼143,000 colonies. These six came from four starting cultures. In two examples, pairs on strains were isolated from the same plate. Characterization of the pairs (AI273–AI274 and AI275–AI276) revealed that they shared the identical mutation within *TAO3*, reflecting an attached pair of pseudohyphal cells that were physically separated when spread on the plate. There are 225 codons in *URA5* vs. 5750 codons combined for the six RAM pathway genes. Based on size alone we expected a 25.6 fold higher frequency of isolation of pseudohyphal strains compared to 5-FOA resistant strains, or 1 in every 57,546 colonies. The isolation of six mutants from 143,000 screened would be unlikely (P<0.03; Poisson distribution). However, if the pairs of identical *tao3* mutants are considered as one event, then four from 143,000 is not statistically significant (P<0.24).

To circumvent bias due to cell separation in the RAM mutants, an alternative measure of mutation was taken. Uracil auxotrophs were isolated in the AI228 mutant, and the *URA5* gene sequenced. AI228 has an a-g transition in an intron splice site in *CBK1*. Strain AI228 ura#3 was isolated with a g-a transition in a highly-conserved glycine codon. We reasoned that the only way to revert the strain to wild type would be the perfect reversal of the mutation. 15 separate colonies were inoculated into liquid medium, cultured overnight, and from each 2.5×10^8^ cells plated onto media supplemented with FK506, to select for a mutation in *CBK1*, and onto media without uracil, to select for a mutation in *URA5*. No wild type yeasts were obtained. In contrast, nine uracil prototrophs were isolated, reflecting reversion in *URA5*. These results indicate that at least one gene in the RAM pathway, *CBK1*, is not a general hot-spot for mutation.

### RAM pathway mutations are associated with phenotypic changes that can be either beneficial or detrimental to survival in the wild

If amoeba select for pseudohyphal strains of *C. neoformans* in the wild, then why are pseudohyphal strains not isolated on a regular basis? We explored this question by testing isolation methods for *C. neoformans* and the fitness of the RAM pathway mutants under different growth conditions.

**Figure 8:**
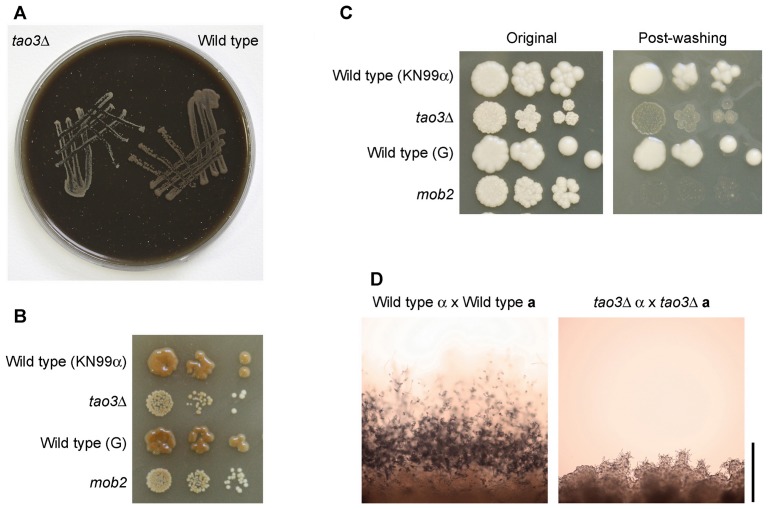
Phenotypes of *C. neoformans* var. *grubii* RAM pathway mutants related to fitness or strain isolation from the environment. **A.** Equivalent growth between two strains (KN99α and AI236) on 10% pigeon guano medium, three days at 30°C. **B.** Growth and melanization of 10-fold serial dilutions on bird seed agar; six days at 22°C. **C.** Colony adherence is reduced in RAM mutants. 10-fold serial dilutions were plated on YPD agar and grown six days at 22°C (left). The plate was washed under running water and photographed (right). **D.** RAM pathway mutants are infertile. Wild type (KN99**a**×KN99α) or *tao3*Δ (AI236×AI257) crosses were mixed on Murashige-Skoog medium, incubated in darkness at room temperature, and the edge of the mix photographed ten days later. Wild type crosses produce a mass of filaments, terminating in chains of basidiospores whereas the *tao3*×*tao3* cross does not. Bar = 500 µm.

First, we explored the ability of RAM pathway mutants to grow on medium that mimics an environmental substrate, pigeon guano, with which *C. neoformans* is associated in nature [47]. Both wild type and RAM mutants grew equally well on pigeon guano medium, suggesting that RAM mutants have equivalent growth as wild type on this substrate (Fig. 8A).

Second, RAM mutants were grown on bird seed agar. This medium is made from *Guizotia abyssinica* seed and is a standard medium for isolation of *C. neoformans* from environmental sources, aided by melanization of *C. neoformans* colonies [48], [49]. In two serotype A genetic backgrounds, the RAM mutant strains were delayed in pigmentation and produced smaller colonies compared to wild type (Fig. 8B). Melanin is a well-established virulence factor for *C. neoformans*. Another virulence trait is the biosynthesis of a polysaccharide capsule, which was found previously to be produced like wild type in RAM pathway mutants [28].

Third, phenotypes were explored under various stress conditions. No visual differences were observed between wild type and pseudohyphal strains growing on YPD at pH 4.5 or pH 8, or on YPD supplemented with high levels of salt NaCl, detergent sodium dodecyl sulfate, antifungal flucoazole, or oxidative stress agents (H_2_O_2_ and *t*-butyl hydroperoxide). One phenotype identified that did differ is altered colony integrity. The RAM pathway mutant cells were easily dispersed by washing (Fig. 8C), possibly a detrimental trait leading to reduced protection of the cells within a structured colony in the wild. Mutation of the *CBK1* homolog in the basidiomycete fungus *Ustilago maydis* causes sterility [33]. *C. neoformans* RAM mutants also have reduced fertility in crosses in which both parents bear mutations, since no filaments, basidia or basidiospores are produced in crosses on V8 juice or Murashige-Skoog medium (Fig. 8D).

Thus, explanations for the lack of pseudohyphal *C. neoformans* isolated from the wild could include their discard due to reduced melanization, reduced growth on bird seed agar or at elevated temperatures, and unconventional cell morphology. Alternatively, while it is possible that RAM mutation confers benefits under some environmental conditions, under others the pseudohyphal strains are less fit thereby countering the advantages gained in avoiding predation by amoeba.

The mechanism by which the RAM mutants of *C. neoformans* evade amoeba was explored using light microscopy, with confocal microscopy of GFP-expressing fungal strains and amoeba stained with FM4–64 used to confirm internalization of the fungal cells (data not shown). These results indicated that one possible mechanism of action is less efficient phagocytosis of RAM pathway mutants by amoeba. The pseudohyphal strains, especially when in clusters of cells, are larger than the amoeba thereby forming a physical impediment to phagocytosis (Fig. 4B). Consistent with this hypothesis, pseudohyphal cells are less frequently found inside amoeba. For instance, a mixture of wild type and *tao3* mutants was exposed to amoeba. Only 3% of amoeba that harbored *Cryptococcus* had cells that were pseudohyphal (*n* = 178). In the cases in which pseudohyphal cells were present in amoeba, these were as single or two attached cells (Fig. 4B). While a physical block may account in part for evasion of amoeba, other reasons for resistance, such as increased intracellular survival of pseudohyphal cells, cannot be excluded.

### A RAM pathway mutant isolated by exposure to amoeba is attenuated for virulence, unless it reverts to wild type during the course of the infection

Based on previous observations of the virulence of pseudohyphal *C. neoformans* and the role of the RAM pathway in virulence in other fungi, we hypothesized that our mutants would be attenuated or avirulent. Wild type strain G, the *mob2* mutant strain DM09 that was isolated by exposing strain G to amoeba, and a complemented strain AI255 (DM09+*MOB2-NEO*) were used to test the role of the RAM pathway in virulence in wax moth larvae and mouse models.

The *mob2* mutant strain DM09 exhibits reduced growth at 37°C, which is predicted to influence virulence in mammalian hosts. To address virulence at a more permissive temperature, the three strains were inoculated into wax moth (*Galleria mellonella*) larvae and the larvae maintained at 30°C. 1×10^5^ cells of wild type and complemented strains were used as inocula. Two inocula were used for DM09: 1×10^5^ cell-clusters and one at 1/10 that concentration. Microscopic analysis of the cultures of the *mob2* mutant and accompanying plating assays indicated that approximately 10 pseudohyphal cells formed the equivalent of one colony forming unit, due to the cell separation defect of the strain.

**Figure 9:**
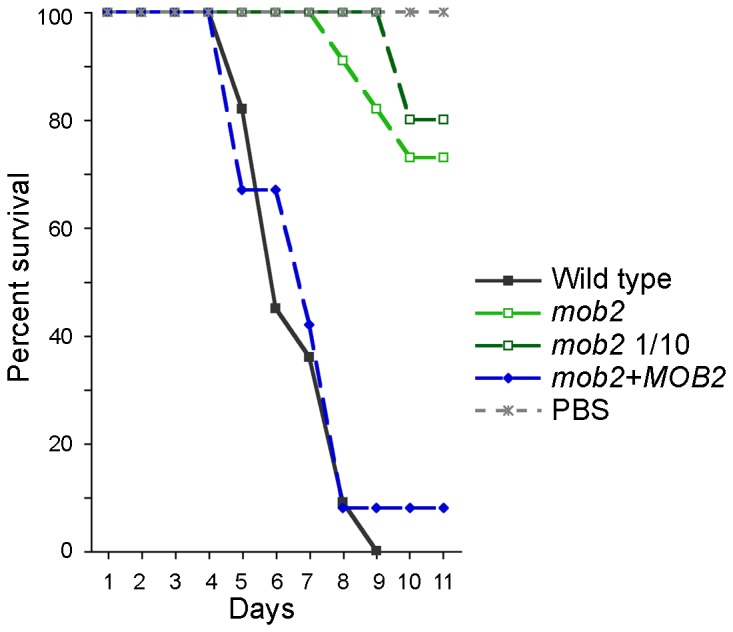
RAM pathway mutants are attenuated for virulence in wax moth larvae. Survival of wax moth larvae infected with wild type strain G (*n* = 11), *mob2* mutant DM09 at two concentrations (*n* = 11 of 1×10^5^ cfus, *n* = 10 of 1×10^4^ cfus), complemented strain AI255 (*n* = 12), and phosphate buffered saline control (*n* = 11).

The larvae inoculated with wild type or complemented strains started dying five days after inoculation, and 22 of 23 were dead by day nine (Fig. 9). In contrast, the larvae inoculated with the *mob2* mutant strain survived longer, e.g. on day nine only two of the 21 larvae had died. The experiment was terminated when the surviving larvae, including the control group inoculated with PBS, formed cocoons. Log-rank statistical comparisons indicated that the differences in survival between wild type or complemented strains with the *mob2* mutant were significant (*P*<0.0001). Thus, the RAM pathway controls fungal virulence in an insect model and reduced virulence is independent of temperature.

**Figure 10:**
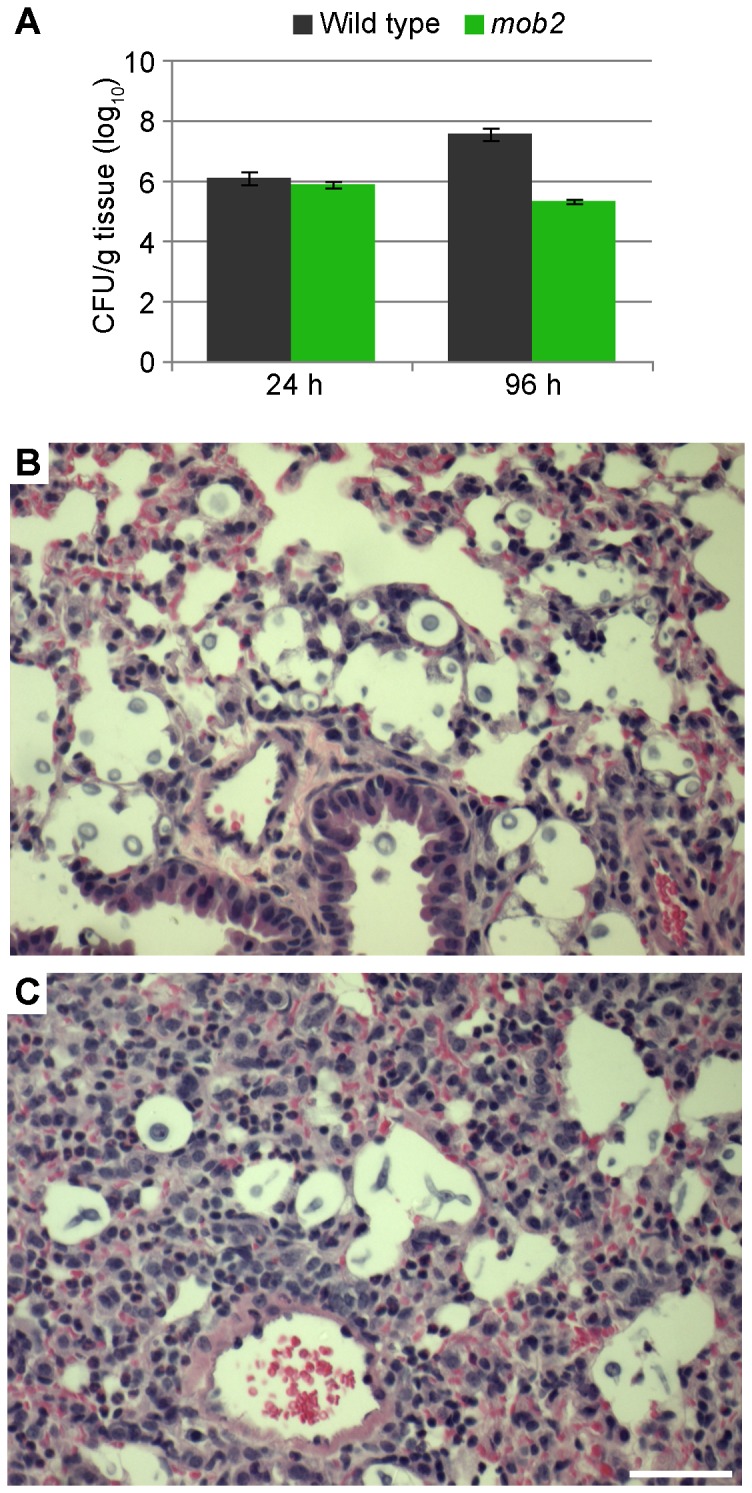
RAM pathway mutants can establish an infection within mice. **A.** Cell counts 24 and 96 h post-inoculation with wild type strain G or *mob2* mutant strain DM09. Three mice were used for each time point and strain, expect *mob2* 24 h in which two mice were used. Each error bar indicates the standard error of the mean. **B.** and **C.** H&E stained lung tissue from mice sacrificed 96 h after inoculation with wild type or the *mob2* mutant strains. Bar = 50 µm.

Next, the three strains were tested in a mouse inhalation model of cryptococcosis. A subset of mice were sacrificed 24 and 96 h post-inoculation, and colony counts measured and lung tissue prepared for histology. The wild type proliferated during the three day interval, while the *mob2* mutant strain maintained a level of ∼1×10^5^ cfu per gram (Fig. 10A). The colonies isolated from mice inoculated with the wild type were smooth and comprised of yeast cells. Colonies from mice inoculated with the *mob2* mutant were all wrinkled and comprised of pseudohyphal cells. Yeast or pseudohyphal cells were evident in histological samples of the lungs at both 24 and 96 h from mice infected with the wild type and *mob2* mutant, respectively (Fig. 10B, C). These results show that *C. neoformans* pseudohyphal cells can penetrate the lung and survive at least four days.

**Figure 11:**
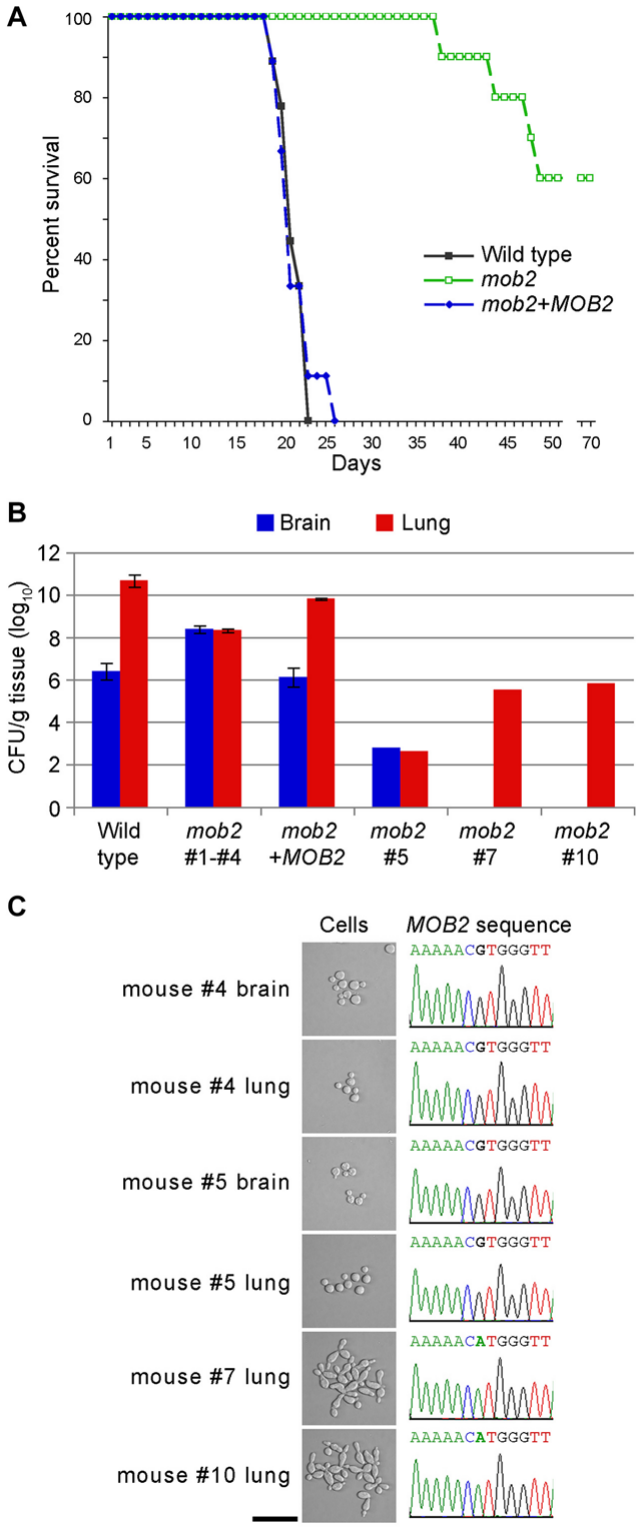
RAM pathway mutants are attenuated for virulence, and can only cause disease if they revert to wild type in a mouse model. **A.** Survival of mice infection with wild type strain G (*n* = 9), *mob2* mutant DM09 (*n* = 10) and complemented strain AI255 (*n* = 9). **B.** Colony forming units measured from brain and lung tissue of mice when sacrificed. For the wild type and complemented strain, three mice were used. For the *mob2* mutant, all ten mice were examined. Four (#1–#4) that caused disease symptoms were sacrificed. The other six were sacrificed at day 70. Three had cleared the infection (not on graph), while three had the fungal burdens indicated. **C.** Cell morphology and *MOB2* chromatograms and sequences from strains isolated from mice infected with the *mob2* mutant strain (bar = 50 µm). The *mob2* mutant carries the g-a mutation that impairs splicing. Strains from mice #4 and 5 have a reversion mutation, while strains from mice #7 and #10 are pseudohyphal and maintain the original mutation. Sequence data from additional strains is provided as dataset S3.

The remaining sets of inoculated mice were monitored daily for signs of cryptococcal disease. The mice infected with the wild type or complemented strains succumbed to disease and were sacrificed by day 26, a time at which all of the mice inoculated with the *mob2* mutant strain were alive and healthy. Interestingly, four of these *mob2*-inoculated mice developed symptoms of cryptococcosis and were sacrificed between days 38 and 49 post-inoculation (Fig. 11A). Cells isolated from either the lungs or brains of these mice were the round yeast morphology like wild type strains (Fig. 11C). The *MOB2* gene was amplified from two colonies isolated from each organ. All sequences showed the original g nucleotide found in the wild type gene sequence (Fig. 11C, dataset S3). Histological examination of the lungs and brain of the diseased animals also revealed yeast cells, as well as extensive host tissue damage (Fig. S2). The interpretation of these data is that the *mob2* mutation had reverted to wild type in the four animals during the course of infection.

At day 70, the remaining six mice inoculated with the *mob2* mutant had no symptoms of disease, so were sacrificed and organ homogenates plated. Three mice had cleared the infection since they had no fungal cells present in either lung or brain tissue. One mouse (number 5) had wild type morphology cells in the brain and lung (Fig. 11B,C). Sequence of *MOB2* from these cells showed the wild type gene sequence. Two mice (numbers 7 and 10) had pseudohyphal strains only in the lung, and the brains were free of fungal cells (Fig. 11B,C). When the *MOB2* alleles in the pseudohyphal strains were examined by DNA amplification and sequencing, those strains contained the original splicing mutation in *MOB2* (Fig. 11C, dataset S3).

In summarizing the results from the mouse model, the *mob2* mutant was attenuated for virulence (Log-rank test *P*<0.0001). However, the pseudohyphal cells can persist during infection, and reversion mutations occur stochastically over time to restore cell shape to the wild type and fully pathogenic form.

## Discussion

In this study we describe a new mechanism for microevolution in the human pathogenic fungus *C. neoformans* that controls the host range of the organism. *Cryptococcus* species have plastic genomes, with experiments showing changes in chromosome length over time, microevolution during human infection and in culture, and changes in chromosome numbers conferring azole drug resistance [9], [10], [45], [50], [51]. Microevolution modulates the polysaccharide capsule composition that surrounds the cell: this is the best-studied aspect about microevolution in the fungus [44], [52], [53].

The *Cryptococcus* genus is found in association with trees, soil, bird excreta and additional environments that are also homes to other microbial species [1], [14]. One hypothesis is that selection for traits that defend against small predators, such as amoeba or nematodes, has led to species capable of causing disease in humans [13], [14], [15], [16]. The evidence for amoeba-*C. neoformans* interactions date to over half a century ago with the work of Castellani, who showed that the species later named *Acanthamoeba castellanii* could kill *C. neoformans* cells [20]. In the 1970s, amoeba were again co-isolated with *C. neoformans* [15], [17]. Subsequent studies found that a subset of *C. neoformans* colonies changed cellular morphology after exposure to *Acanthamoeba* species, from yeast to pseudohyphal cells, and that some isolates reverted rapidly to wild type yeast. Based on the more recent observation of phenotypic switching between pseudohyphal and yeast forms [27] and the discovery of a set of genes that produces a pseudohyphal phenotype when mutated [28], we hypothesized that phenotypic switching involved the RAM pathway.

Here we show that the RAM pathway is the integral component of “switching” in *C. neoformans* because it is mutated in pseudohyphal strains isolated from amoeba and spontaneously in culture. The largest gene in the pathway, *TAO3*, most commonly bears point mutations leading to the introduction of premature stop codons. Some strains like F7 revert to wild type at a high frequency *in vitro*. Analysis of the mutated region in those strains reveals that there are multiple ways in which the strain can revert (Fig. 5). This may be through a bp substitution leading to a coding triplet being reformed. Alternatively, the stop codon may still be present. The basis for the latter situation is unknown. It could be mediated by stop codon read through, tRNA suppressor mutations, changes in downstream gene expression, or epigenetic phenomena. Mutation in another RAM pathway component may suppress the phenotype, as occurs with a specific residue in the Cbk1 kinase of *S. cerevisiae* to rescue mutations in other pathway components [54], or be modified by interacting pathways such as seen for the *Neurospora crassa cot-1* suppressors [55], [56]. An alternative mechanism of reversion is illustrated by strain DM03 bearing a mutation in *SOG2*. Excision of the duplicated region or deletion of another region downstream reverts Sog2 sequence back to wild type or in frame, respectively. Taken together, the conversion between yeast and pseudohyphal cells reported previously as a form of phenotypic switching is based on DNA mutations, rather than epigenetic changes.

The effect of a defective RAM pathway on mammalian virulence was tested. The *mob2* mutant strain used carries a bp substitution mutation within an intron splice site, and is phenotypically stable. Three strains were inoculated into mice. The wild type and complemented strains caused cryptococcal disease. In contrast, different outcomes were observed for mice infected with the *mob2* mutant (Fig. 11). Four mice succumbed to disease, albeit weeks after those infected with the wild type and mutant had been sacrificed, and when their organs were harvested only yeast cells were recovered rather than the expected pseudohyphal cells that were used to inoculate the animals. The *MOB2* gene was sequenced, and now had the wild type sequence (Fig. 11C). Sacrifice of the remaining and asymptomatic animals at day 70 and characterization of fungal material in lung and brain tissue shows that three mice had cleared the infection, one carried wild type cells, and the other two still maintained the original *mob2* mutant phenotype and genotype. These results are consistent with previous virulence studies using pseudohyphal strains selected by amoeba in which reversion back to wild type for some occurred at a high frequency within mouse models [17], [25], [26]. A third animal experiment has been performed with pseudohyphal strains, presumably also RAM mutants, in a rat tracheal model [27]. In this experiment, two of the four animals cleared the infection while the other two did not, potentially representing another case of reversion within the host.

Morphological differentiation is important for *Cryptococcus* pathogenesis. Recently, a role has been assigned for a giant cell form during disease development [57], [58], while constitutive filamentation by altered regulation of the *ZNF2* gene impairs virulence [59]. There are reports of pseudohyphal cells in histopathological samples of patients infected with *C. neoformans* [60], [61]. One speculation is that pseudohyphal forms could allow escape of the fungus from mammalian or amoeba cells, in addition to escape of yeast cells from macrophages or *A. castellanii* by exocytosis [62], [63], [64].

How can “switching” occur at high frequency? The formation of pseudohyphal strains relates to mutation rates in cells; switching also increases upon exposure to UV light [27]. Two factors influence frequency. The first is that the strain used in phenotypic switching studies has a 25 times higher mutation rate compared to a standard wild type strain. Second, because there are six genes in the RAM pathway there is a large amount of target DNA available for spontaneous mutation. Protein-coding sequence alone, the six genes comprise more than 17 kb of DNA, or ∼0.1% of the genome (Fig. 2). *TAO3*, as the largest member (42% of total cumulative size), is therefore the most likely gene to be hit. The original historical isolates bear mutations in this gene. Of 14 unique mutations that we defined in the ATCC 24067A background, eight are in *TAO3*, supporting this hypothesis. One challenge for quantitative analysis of the pseudohyphal strains is their defect in cell separation. Reversion to wild type has been estimated as high as 1.6×10^−3^ [27], but this may be an over-estimate by up to an order of magnitude if the colony forming units were derived from attached cells.

It is unknown why an organism like *Cryptococcus*, which is normally found in the environment, can cause disease in humans or other mammals. Further, the fungal behavior upon entering the human host that ends in life-threatening disease remains unclear. Three points are worth raising. First, people are exposed to *C. neoformans* during their lifetimes yet most do not develop disease. For instance, children in city environments where there is a high prevalence of pigeons as sources of *C. neoformans* become antigen positive at an early age [65]. Second, cases of re-activation from quiescent infections brought about by immunosuppression supports a hypothesis that the fungus enters a latent state [66]. Third, in comparing virulence of strains derived directly from the environment vs. a human host, many environmental strains do not cause disease in animal models although they persist in the lungs [67], [68], [69], [70]. Collectively, a high rate of exposure to *C. neoformans*, to strains that may not necessarily be able to cause disease immediately, and the potential for latency provide the scenario in which microevolution of *C. neoformans* by DNA mutations could influence clinical outcomes.

Multiple mechanisms can facilitate microevolution in pathogens. Among the fungi, mutations during infection lead to the emergence of antifungal drug resistance. However, these arise under conditions with a high fungal burden in the host, promoting the generation of strains from rare events. More broadly, there is evidence from diverse microbes that DNA is mutated to generate phenotypic variation. In the protist *Trypanosoma brucei*, mutation is required for evasion of the host immune response whereby double stranded breaks are generated and then repaired to generate antigenic diversity via the *VSG* genes [71]. Adaptation via mutation is implicated in bacterial disease progression. For example, 20% of *Pseudomonas aeruginosa* isolates from cystic fibrosis patients are “hypermutators” compared with 0% of environmental isolates [72]. Nevertheless, this trend in bacteria is not universal, as correlations between clinical isolates of *E. coli* and higher mutation rates have been found in some, but not all, studies [73], [74]. A very different system to develop variation is the low fidelity of the human immunodeficiency virus' reverse transcriptase: along with other factors this results in rapid genetic changes within the human host and reduces immunological recognition of the virus. In contrast, evidence for mutations affecting the pathogencity of *C. neoformans* are rare at present. Curiously, a *C. neoformans* mutant in the *MSH201* gene predicted to function in mismatch repair has a competitive advantage in mouse lungs compared to control strains [75].

This research points towards future directions into investigating the contribution mutation and mutation rates play in the ability of *C. neoformans* and other pathogenic fungi to adapt and cause disease. Specific directions are to assess mutation rates within the host and test if mutations that arise in the host result in more virulent strains. Second is to test for correlations between clinical and environmental isolates and rates of mutation. A third direction is to explore what role, if any, transcriptional, translational or epigenetic regulation of the RAM pathway plays in the interaction of *C. neoformans* with amoeba and mammalian cells.

## Materials and Methods

### Strains and growth conditions

*Cryptococcus neoformans* wild type strains used were KN99α (var. *grubii*), G (var. *grubii*, ATCC 42347), ATCC 24067, ATCC 24067A (var. *neoformans*), and JEC21 (var. *neoformans*). ATCC 24067A is derived from laboratory passage of ATCC 24067. Historical pseudohyphal strains were F7 (var. *neoformans*), and C, D, and E (var. *grubii*; ATCC 42343-5). Strains were kindly provided by Dr. Joseph Heitman and Dr. Bettina Fries. *C. neoformans* strains were cultured on yeast extract-peptone dextrose (YPD) ±2% agar medium, and stored as glycerol stocks at −80°C. The *Acanthamoeba castellanii* strain was obtained from the American Type Culture Collection (ATCC 30234) and maintained according to ATCC instructions and stored at 4°C [76]. To isolate new pseudohyphal strains, ATCC 24067A was grown in overnight YPD cultures, then spread on YPD plates. A subset of plates were subject to a low dose of UV light in a UV transilluminator. Colonies were screened by eye for those with a dry appearance, which were streaked to isolate single colonies. To isolate RAM mutants using the *A. castellanii* amoeba, *C. neoformans* strains ATCC 24067A and G were inoculated in a cross pattern on a selection of different agar medium types (potato dextrose, proteose peptone, YPD, V8 juice, Murashige-Skoog and trypan blue), and a drop of amoeba placed at the intersection, following the original protocol [17]. T-DNA insertional mutants were generated as described previously and RAM pathway mutants isolated based on colony morphology [28]. Wild type revertants were selected from RAM pathway mutants by plating on YPD agar supplemented with FK506 (1 µg/ml). Pigeon guano medium was 10% w/v of unfiltered pigeon guano (collected under the I-35 overpass of Southwest Blvd, Kansas City, MO) that had been homogenized in a coffee grinder and autoclaved with 4% agar. Bird seed agar was prepared as described [48]. Crosses were set up on 5% V8 juice or Murashige-Skoog agar [77]. Strains used and generated during this study are listed in Table S1.

### Sequence analysis to identify point mutations

Genomic DNA was extracted using a CTAB buffer [78] from 50 ml overnight cultures of strains. The *TAO3* gene was amplified by PCR with two primer sets for each variety: ALID0013–ALID0061 and ALID0014–ALID0060 for var. *grubii* strains, and ALID0127–ALID0128 and ALID0129–ALID0138 for var. *neoformans* strains. *SOG2* was amplified with primers ALID0123–ALID0162. *MOB2* was amplified with primers DM062–DM063. *CBK1* was amplified with primers ALID0977–ALID0978. Part of the KIC1 gene was amplified with primers ALID1681–ALID1682 The PCR products were sequenced with the primers used for amplification and additional internal primers. Primer sequences used for amplification are listed in Table S2.

### Gene complementation

Tests were performed on pseudohyphal strains using vectors that complement the deletion mutants of *mob2*, *cbk1*, *kic1*, and *sog2*. The first three plasmids were generated in a previous study on the RAM pathway [28]. The *SOG2-NEO1* construct was generated by amplification of *SOG2* from strain JEC21 with primers ALID0123–ALID0162, cloning into TOPO pCR2.1 (Invitrogen, Life Technologies, Grand Island, NY), and a SpeI-XbaI fragment subcloning into the XbaI site of pPZP-NEO11. The four genes were in plasmids enabling their introduction into *C. neoformans* cells via *Agrobacterium*-mediated transformation [79]. Transformants were selected on YPD medium containing cefotaxime (200 µg/ml) and either nourseothricin (100 µg/ml) or neomycin (200 µg/ml). The *TAO3* gene in strain D was reconstituted by homologous recombination. A construct with an engineered BglII restriction enzyme site was generated by overlap PCR using primers ALID0061–ALID0227 and ALID0111–ALID0228, and introduced into strain D cells plated on YPD+1 M sorbitol by biolistic transformation with a PDS-1000/He Particle Delivery System (Bio-Rad, Hercules, CA), using standard methods [80]. Cells were allowed to recover for 3 h and transferred to YPD+FK506 (1 µg/ml).

### Deletion of *SOG2* and *TAO3*

The *TAO3* gene was deleted in the KN99α, ATCC 24067A and G strains. For var. *grubii* strains, the 5′ and 3′ flanks were amplified from genomic DNA of strain KN99α using primers damp5-ALID0013 and damp6–damp7, respectively. For var. *neoformans*, the 5′ and 3′ flanks were amplified from strain ATCC 24067A using primers damp1–damp2 and damp3–damp4, respectively. Nourseothricin acetyltransferase (*NAT*) was amplified from plasmid pAI3 using primers ai006–ai290 [79]. The primers ALID0013-damp7 or damp1–damp4 were used for overlap PCR. The *SOG2* gene was deleted in strain KN99α. Primers ALID0483–ALID0484 and DM036–DM043 were used to amplify the 5′ and 3′ flanks, and ALID0483–DM036 used for overlap PCR with these and the *NAT* cassette. The DNA molecules were transformed into *C. neoformans* cells using the biolistic apparatus, cells allowed to recover for 3 h, and transferred to YPD medium containing nourseothricin (100 µg/ml). Correct gene replacement was confirmed by PCR analysis and Southern blotting with [^32^P]-dCTP-labelled fragments of the genes.

### Mutation rate analysis

Isolation of spontaneous uracil auxotrophs was used to measure mutation frequency. For comparison between strains ATCC 24067, which was acquired from the ATCC, and ATCC 24067A, strains were grown on yeast nitrogen base (YNB) medium, then 20 separate cultures of each strain established at 1×10^5^ cells/ml in YPD medium. After overnight culture in a roller drum incubated at room temperature, 5×10^7^ or 1×10^8^ cells were plated onto YNB supplemented with uracil (20 mg/L) and 5-fluoroorotic acid (5-FOA; 1 g/L) medium. The resulting colony numbers were analyzed to determine the mutation rate (Lea-Coulson method of the median) with FALCOR software [81]. To ensure mutations targeted the same gene in both strains, the *URA5* gene was amplified with primers ALID0375–ALID0376 and sequenced.

To compare mutation rates in *URA5* to the RAM pathway genes, aliquots from the same 20 ATCC 24067A cultures used to isolate *URA5* mutants were diluted and plated onto ten YPD plates. Dry colonies were screened visually and pseudohyphal morphology confirmed by microscopy. The nature of the mutation in these strains was identified by complementation tests and DNA sequence analysis. Statistical analysis used the Poisson distribution, testing the probability of isolating *n* or more pseudohyphal strains. Strain AI228 ura#3 (*cbk1 ura5*) was inoculated into 15 YPD cultures, grown overnight, and 2.5×10^8^ cells plated onto YPD+2 µg/ml FK506 and YNB.

### RNA purification and northern blotting

50 ml cultures in liquid YPD medium were incubated overnight at 150 rpm at room temperature. The cells were frozen and lyophilized. Total RNA was extracted with TRIzol (Invitrogen) or TRI reagent (Sigma-Aldrich, St. Louis, MO). For northern blots, 10 µg of RNA purified from each strain were resolved on 1.4% agarose/formaldehyde gels. RNA was blotted to Zeta-Probe membrane (Bio-Rad). [^32^P]-labeled probes of the six RAM genes (the primers used for amplification are in Table S2) were hybridized to blots. Blots were stripped and reprobed with actin (*ACT1*) as a loading and RNA transfer control. RNA purified from wild type strain KN99α was also used to confirm the intron-exon boundaries of *TAO3*, by sequencing cDNAs reverse transcribed with Superscript III (Invitrogen).

### Virulence studies in wax moth larvae and mice

Wax moth larvae (*G. mellonella*) were purchased from Vanderhorst Wholesale (Saint Mary's, OH). Overnight cultures of *C. neoformans* grown in liquid YPD were washed in phosphate buffered saline (PBS), the concentration determined by counting cells with a hemocytometer, and diluted such that 1×10^5^ cells of wild type and the complementation *mob2*+*MOB2* strain were injected into the larvae as described previously [82]. For *mob2* mutant strain DM09, 1×10^5^ and 1×10^6^ cells were inoculated. Concentrations were confirmed by plating serial dilutions onto YPD agar plates.

Groups of female A/JCr mice (NCI-Frederick, MD) were infected intranasally with 10^5^ cfus of each strain, as previously described [83]. Inocula were confirmed by plating onto YPD agar. Animals that appeared moribund or in pain were sacrificed by CO_2_ inhalation. For cfu assays, lungs and brain were dissected from animals, homogenized in PBS, and plated onto YPD medium containing ampicillin and chloramphenicol. Colonies were determined after incubation for 3 d at 30°C. For histology, lung and brain samples were fixed and hematoxylin and eosin (H&E) stained. Survival data from the murine experiments were statistically analyzed between paired groups using the log-rank test in the PRISM program 4.0 (GraphPad Software). P values of <0.01 were considered significant.

### Ethics statement

The mouse experiments were performed in full compliance with a protocol approved by the University of Medicine and Dentistry of New Jersey Institutional Animal Care and Use Committee, and in compliance with the United States Animal Welfare Act (Public Law 98–198). The experiments were carried out in facilities accredited by the Association for Assessment and Accreditation of Laboratory Animal Care.

### GenBank accessions

Sequences of *CBK1*, *MOB2* and *TAO3* from strain ATCC 24067A have been deposited to GenBank, under accessions HM770879, JX297541 and GU903010, respectively.

## Supporting Information

Dataset S1 Nature of the RAM mutations in strains identified during this study
(PDF)

Dataset S2 Nature of the *URA5* mutations in 5-FOA resistant isolates from *C. neoformans* strains ATCC 24067 and ATCC 24067A.
(PDF)

Dataset S3 *MOB2* sequences from strains recovered from mice inoculated with strain DM09. The *MOB2* gene was amplified from two single colonies isolated from each tissue type, and the region covering the mutation present within strain DM09 sequenced. The g-a mutation in DM09 abolishes an intron splice site (intron in lower case), leading to introduction of a premature stop codon (TAA).
(PDF)

Figure S1 Northern blot analysis of RAM pathway genes in wild type and RAM mutant strains grown in liquid yeast extract-peptone-dextrose liquid medium. 10 µg of total RNAs, resolved in denaturing agarose gels and transferred to Zeta-Probe membrane, were probed with the six RAM pathway genes and actin (*ACT1*). Insufficient signal was detected for *KIC1* and *TAO3*.
(TIF)

Figure S2 Histology samples of lung (**A**) or brain (**B**) from mouse #3 that was infected with *mob2* mutant strain DM09 and sacrificed due to exhibiting signs of disease (H&E stained; bar = 50 µm). The pseudohyphal trait has reverted to the wild type yeast morphology.
(TIF)

Table S1 *C. neoformans* strains used in this study. A subset of the *in vitro* revertants is listed.
(PDF)

Table S2 Oligonucleotide primers used for amplification of DNA.
(PDF)
