# Supplementary material for: DNA Mutations Mediate Microevolution between Host-Adapted Forms of the Pathogenic Fungus Cryptococcus neoformans
Source: PLoS Pathog. 2012 Oct 4;8(10):e1002936. doi: 10.1371/journal.ppat.1002936 (PMC3464208; doi:10.1371/journal.ppat.1002936)
Supplement: Dataset S1 — Nature of the RAM mutations in strains identified during this study (PDF) [file ppat.1002936.s001.pdf]

C. neoformans **var.** ***neoformans*** **strain ATCC 24067A**

TAO3

```
WT      GACGCTGGTGGGAACAATTAAAAAGGAAGGGAACAAATTG
F7      GACGCTGGTGGGAACAATTATAAAGGAAGGGAACAAATTG
Consequence: premature stop codon

WT      TCTCAAAAGGTGTGGTCCTTTATTCCTCAACACCTTGGTA
DM01    TCTCAAAAGGTGTGGTCCTTAATTCCTCAACACCTTGGTA
Consequence: premature stop codon

WT      ATTCGACAGCATCGTCACTT--CTCTCTCACAATGCGCAACG
DM02    ATTCGACAGCATCGTCACTTTTCTCTCTCACAATGCGCAACG
Consequence: frame shift

WT      AGAGCATATTGTAGAGCCATTATTAGTATTGAGGTGGATAA
DM04    AGAGCATATTGTAGAGCCATAATTAGTATTGAGGTGGATAA
Consequence: premature stop codon

WT      TTTCAAACTGTGTACCGAATGGTGTTTGGTCGGGCGTCGGC
DM05    TTTCAAACTGTGTACCGAATAGTGTTTGGTCGGGCGTCGGC
Consequence: premature stop codon

WT      ATACTGACTCTTATGGTCATCGAACGAATCTCTCTTCCGAC
DM11    ATACTGACTCTTATGGTCATTGAACGAATCTCTCTTCCGAC
Consequence: premature stop codon

WT      CCTCTGACTCAAGCCCCGCACAACAAGCGTCAGAGCATATT
DM12    CCTCTGACTCAAGCCCCGCATAACAAGCGTCAGAGCATATT
Consequence: premature stop codon

WT      GACATGCGCGGGGCCATTTT-CAAACTGTGTACCGAATGGT
AI273   GACATGCGCGGGGCCATTTTTCAAACTGTGTACCGAATGGT (same in AI274)
Consequence: frame shift

WT      TAGTGTTCAAGGAGTTTAGAGAAATGGCCGGTAATAAAGTG
AI275   TAGTGTTCAAGGAGTTTAGATAAATGGCCGGTAATAAAGTG (same in AI276)
Consequence: premature stop codon
```

SOG2

```
WT      CGCGGCGCGAGCTATACTCC----------------TGCACAACGCAACTCTGGGC
DM03    CGCGGCGCGAGCTATACTCCTGCACAACGCAACTCTTGCACAACGCAACTCTGGGC
Consequence: frame shift
```

CBK1

```
WT     cggttcattgactattgtttagGATGCTTTGCGAGGCTGAC
AI288  cggttcattgactattgtttggGATGCTTTGCGAGGCTGAC
Consequence: impaired intron splicing
```

MOB2

```
WT     TTCAGTCTAAACTTCCTTTGGCCAGATCACAACCAGAGACT
DM13   TTCAGTCTAAACTTCCTTTGACCAGATCACAACCAGAGACT
Consequence: premature stop codon
```

```
WT     CATCTTTACCATGCCCATTTCGAGCAAGTCCTCCACCTCTC
AI237  CATCTTTACCATGCCCATTT-GAGCAAGTCCTCCACCTCTC
Consequence: frame shift
```

KIC1

```
WT     catctcctaacaccaagcaaagTCTCGGGCACAACCACTGA
AI277  catctcctaacaccaagcaatgTCTCGGGCACAACCACTGA
Consequence: impaired intron splicing
```

```
WT     tctatatttgaacacgcctaatctccctcccattcttagCT
AI278  tctatatttgaacacgcctagtctccctcccattcttagCT
Consequence: new intron splice site
```

C. neoformans var. *grubii* strains G or KN99α

TAO3

```
WT      CAGGTGAAATCTTGCAATTCGAAGTAGAATCAAAAGCGGAA
D       CAGGTGAAATCTTGCAATTCTAAGTAGAATCAAAAGCGGAA
Consequence: premature stop codon

WT      AGCATTGGTCGGTCACCAACGCGTAGCAGAGGCACAAGCTCAAGCAG
DR3     AGCATTGGTCGGTCACCAAC-------AGAGGCACAAGCTCAAGCAG
Consequence: frame shift

WT      CTTACGTATTAAGGTGGAGGCTGCCCAGTTGTCCGACGCGTTCGAGGCACGGAGGAAATGTCCG
RM2     CTTACGTATTAAGGTGGAGG-------- T-DNA ---------AGGCACGGAGGAAATGTCCG
Consequence: insertion in gene promoter

WT               CGGGCGACATTTTCTGTTGACCCAAAGCTTTGTGCGTCAG
AI227   CGGTCCGATTGCTACTGACA- T-DNA -CCCAAAGCTTTGTGCGTCAG
Consequence: insertion within coding region and a t(2;14) chromosomal
rearrangement

WT      TTTGTTCCACTCTTCCCCAACATCACCACTTTACCCTTCAGA
DM10    TTTGTTCCACTCTTCCCCAAC-TCACCACTTTACCCTTCAGA
Consequence: frame shift
```

KIC1

```
WT      CTGTAGCGATGTGTTTTCCT---------CTGTACACTGCGCCGTACGC
AI217   CTGTAGCGATGTGTTTTCCT- T-DNA -CTGTACACTGCGCCGTACGC
Consequence: insertion within coding region
```

MOB2

```
WT      ACGATGTCTGGTGGAAAAACgtgggttattgtgtttcatat
DM09    ACGATGTCTGGTGGAAAAACatgggttattgtgtttcatat
Consequence: impaired intron splicing
```
