# Supplementary material for: DNA Mutations Mediate Microevolution between Host-Adapted Forms of the Pathogenic Fungus Cryptococcus neoformans
Source: PLoS Pathog. 2012 Oct 4;8(10):e1002936. doi: 10.1371/journal.ppat.1002936 (PMC3464208; doi:10.1371/journal.ppat.1002936)
Supplement: Dataset S2 — Nature of the URA5 mutations in 5-FOA resistant isolates from C. neoformans strains ATCC 24067 and ATCC 24067A. (PDF) [file ppat.1002936.s002.pdf]

Strain ATCC 24067

ATGTCCTCCCAAGCCCTCGACTCCGCCAAAGTTGCCTTCATTGAGGCTGCCATCGAACATGGCGTGCTTCTTTTCGG
CAACTTTACCTTGAAGTCCGGCCGgtgagccatattgcagcgcttcacagtccaatcgaatctgacatgtgttcagT
CAATCCCCTTACTTCTTCAATGCCGGTCTCCTTTA**C**TCTTCAT**C**GCTTCTCTCAACTACCGCTCAGGCTTACGCCAA
GATACTTTCCTCTTCTAGGATTCCTGACTTTGACGTCCTCTTCGGCCCAGCTTACAA**G**GGTAT**|**CTCCTTGGCTGCT
GTCTCCGCTGTAAGCCTTTATCAG**C**AAACCGGCAAAGATATCGGCTACTGCTACAACAGGAAGGAGAAGAAGGACg**t**
gagtctgtcccaaccagtgcgacagcgatgagctcataagccagtagCACGGTGAGGGCGGTACTATGGTCGGTGCG
CCTCTCAAGGGACGAATCGTCATCATCGACGATGTTCTCACCTCTG**G**CAAGGCCATCCGTGAAGCTATTGACATTCT
CAAGGCCTCCCCTGAAGCGAAGCTTGTCGG**AATTGTCCAGCTTGTCGA**CAGACA**AGA**GAAAGGCCAGAGCGGTAGCG
GCAAGAGTACCGTACAGGAGGTTGAGGAAGAGTTCGGTGTGCCTGTCGAGCCTATTATTGGTTTGGACGACATTGTG
AAGTACTTAGAAAGCTCCGGCAAGTGGGAAAAGGAGCTGCAAGAGGTCAGGAAGTACAGGGCGGAGTACGGTGTTCA
GAGGTCTTAA

C-T #15
C-A #19
C-A #17
G-T #12
|+AT #3
C-T #8
t-c #4, #9, #18
G-A #5
ATTGTCCAGCTTGTCGA-Δ #2
AGA- Δ #14
Insertion/rearrangement #11, #13, #20

Strain ATCC 24067A

ATGTCCTCCCAAGCCCTCGACTCCGCCAAAGTTGCCTTCATTGAGGCTGCCATCGAACATGGCGTGCTTCTTTTCGG
CAACTTTACCTTGAAGTCCGGCCGgtgagccatattgcagcgcttcacagtccaatcgaatctgacatgtgttc**a**gT
CAAT**C**CCCTTACTTCTTCAATGCCG**G**TCTCCTTTACTCTTCATCGCTTCTCTCAACTACCGCTCAGGCTTACGCCAA
GATACTTTCCTCTTCTAGGATTCCTGACTTTGACGTCCTCTTCG**G**CCCAGCTTACAAGGGTATCTCCTTGGCTGCTG
TCTCCGCTGTAAGCCTTTATCAG**C**AAACCGGCAAA**G**ATATCGGCTACTGCTACAACAGGAA**G**GAGAAGAAGGACg**t**
gagtctgtcccaaccagtgcgacagcgatgagctc**a**taagccagtagCACGGTGAGGGCGGTACTATGGTCGGTGCG
CCTCTCAAGGGACGAA**T**CGTCATCATCGACG**A**TGTTCTCA**C**CTCTGGCAAGGCCATCCGTGAAGCTATTGACATTCT
CAAGGCCTCCCCTGAAGCGAAGCTTGTCGGAATTGTCCAGCTTGTCGACAGACAAGAGAAAGGCCAGAGCGGTAGCG
GCAAGAGTA**C**CGTACAGGAGGTTGAGGAAGAGTTCGGTGTGCCTGTCGAGCCTATTATTGGTTTGGACGACATTGTG
AAGTACTTAGAAAGCTCCGGCAAGTGGGAAAAGGAGCTGCAAGAGGTCAGGAAGTACAGGGCGGAGTACGGTGTTCA
GAGGTCTTAA

a-g #14, #15
C-A #12
G-A #9
G-A AI228 ura#3
C-T #8
G-Δ #2
G-T #11
t-c #1, #7, #10
a-g #5
T-A #4
A-T #13
C-T #6
C-Δ #3
