# Supplementary material for: DNA Mutations Mediate Microevolution between Host-Adapted Forms of the Pathogenic Fungus Cryptococcus neoformans
Source: PLoS Pathog. 2012 Oct 4;8(10):e1002936. doi: 10.1371/journal.ppat.1002936 (PMC3464208; doi:10.1371/journal.ppat.1002936)
Supplement: Dataset S3 — MOB2 sequences from strains recovered from mice inoculated with strain DM09. The MOB2 gene was amplified from two single colonies isolated from each tissue type, and the region covering the mutation present within strain DM09 sequenced. The g-a mutation in DM09 abolishes an intron splice site (intron in lower case), leading to introduction of a premature stop codon (TAA). (PDF) [file ppat.1002936.s003.pdf]

Wild type	ACGATGTCTGGTGGAAAAACgtgggttattgtgtttcatatggtataa. . .
DM09	ACGATGTCTGGTGGAAAAAC**A**TGGGTTATTGTGTTTCATATGGTATAA
Mouse 3 brain 1	ACGATGTCTGGTGGAAAAACgtgggttattgtgtttcatatggtataa
Mouse 3 brain 2	ACGATGTCTGGTGGAAAAACgtgggttattgtgtttcatatggtataa
Mouse 3 lung 1	ACGATGTCTGGTGGAAAAACgtgggttattgtgtttcatatggtataa
Mouse 3 lung 2	ACGATGTCTGGTGGAAAAACgtgggttattgtgtttcatatggtataa
Mouse 4 brain 1	ACGATGTCTGGTGGAAAAACgtgggttattgtgtttcatatggtataa
Mouse 4 brain 2	ACGATGTCTGGTGGAAAAACgtgggttattgtgtttcatatggtataa
Mouse 4 lung 1	ACGATGTCTGGTGGAAAAACgtgggttattgtgtttcatatggtataa
Mouse 4 lung 2	ACGATGTCTGGTGGAAAAACgtgggttattgtgtttcatatggtataa
Mouse 5 brain 1	ACGATGTCTGGTGGAAAAACgtgggttattgtgtttcatatggtataa
Mouse 5 brain 2	ACGATGTCTGGTGGAAAAACgtgggttattgtgtttcatatggtataa
Mouse 5 lung 1	ACGATGTCTGGTGGAAAAACgtgggttattgtgtttcatatggtataa
Mouse 5 lung 2	ACGATGTCTGGTGGAAAAACgtgggttattgtgtttcatatggtataa
Mouse 7 lung 1	ACGATGTCTGGTGGAAAAACATGGGTTATTGTGTTTCATATGGTATAA
Mouse 7 lung 2	ACGATGTCTGGTGGAAAAACATGGGTTATTGTGTTTCATATGGTATAA
Mouse 10 lung 1	ACGATGTCTGGTGGAAAAACATGGGTTATTGTGTTTCATATGGTATAA
Mouse 10 lung 2	ACGATGTCTGGTGGAAAAACATGGGTTATTGTGTTTCATATGGTATAA
