# Supplementary figures and images for: DNA Mutations Mediate Microevolution between Host-Adapted Forms of the Pathogenic Fungus Cryptococcus neoformans
Source: PLoS Pathog. 2012 Oct 4;8(10):e1002936. doi: 10.1371/journal.ppat.1002936 (PMC3464208; doi:10.1371/journal.ppat.1002936)

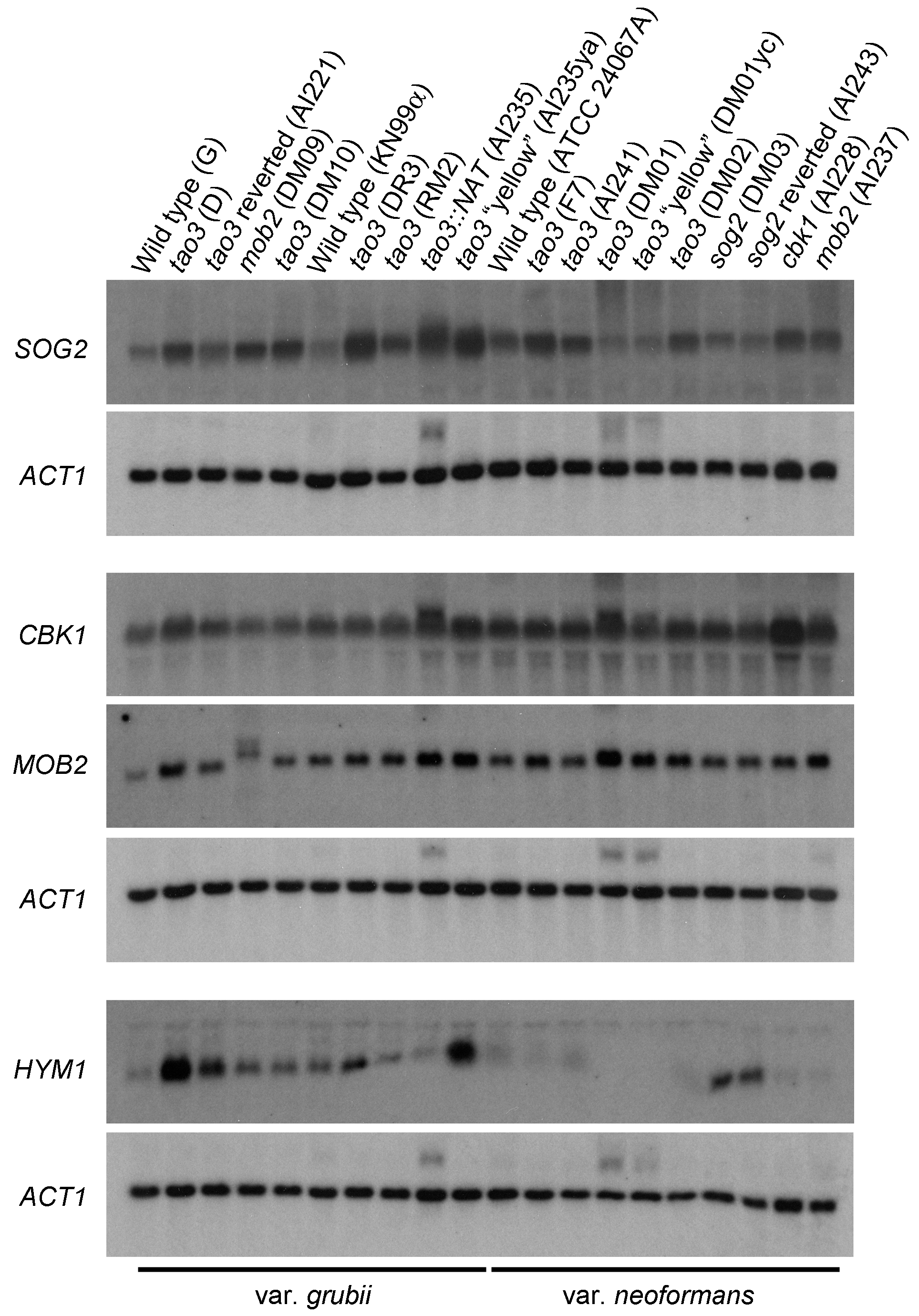

Supplement: Figure S1 — Northern blot analysis of RAM pathway genes in wild type and RAM mutant strains grown in liquid yeast extract-peptone-dextrose liquid medium. 10 µg of total RNAs, resolved in denaturing agarose gels and transferred to Zeta-Probe membrane, were probed with the six RAM pathway genes and actin (ACT1). Insufficient signal was detected for KIC1 and TAO3. (TIF) [file ppat.1002936.s004.tif]

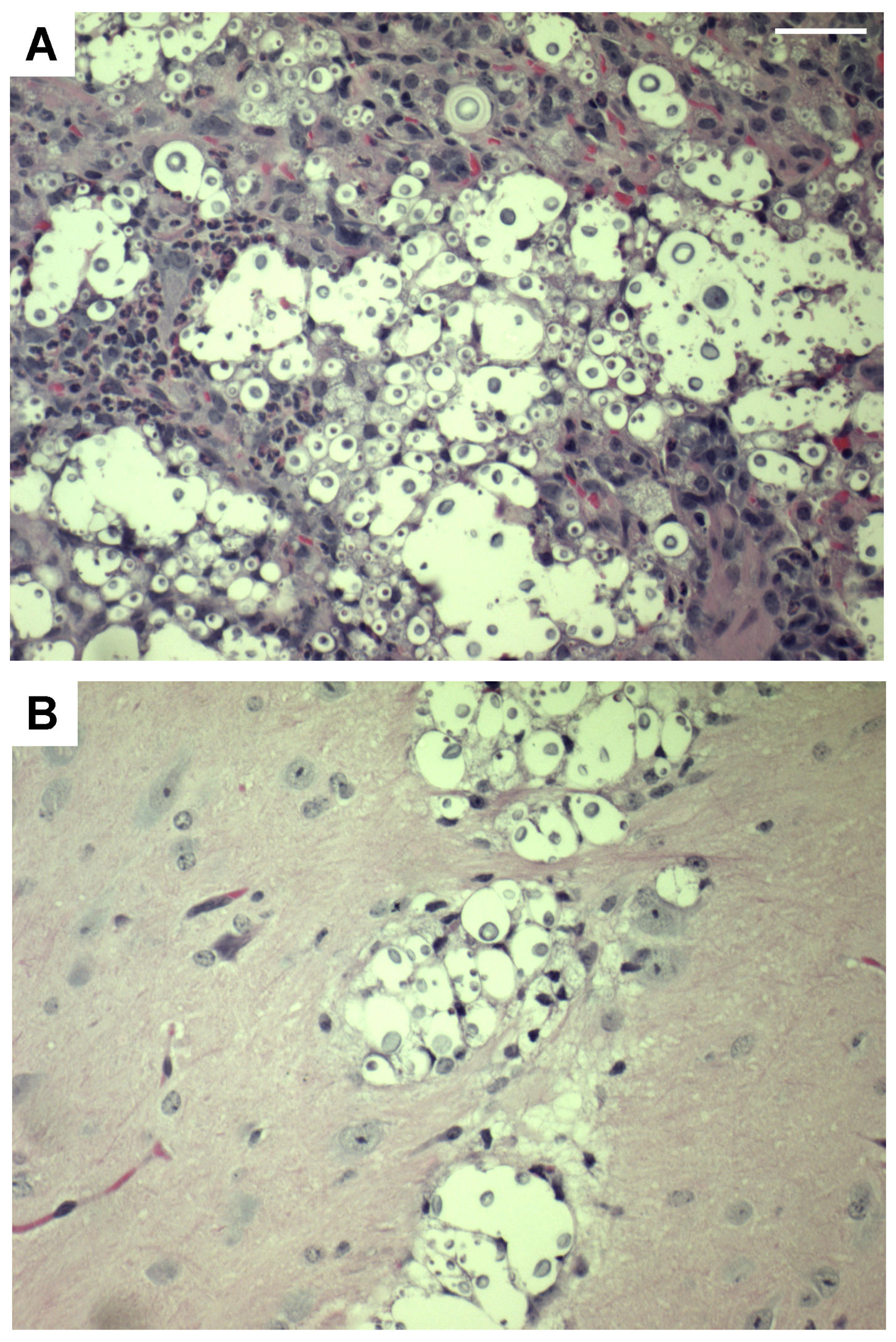

Supplement: Figure S2 — Histology samples of lung (A) or brain (B) from mouse #3 that was infected with mob2 mutant strain DM09 and sacrificed due to exhibiting signs of disease (H&E stained; bar = 50 µm). The pseudohyphal trait has reverted to the wild type yeast morphology. (TIF) [file ppat.1002936.s005.tif]
