# Supplementary material for: DNA Mutations Mediate Microevolution between Host-Adapted Forms of the Pathogenic Fungus Cryptococcus neoformans
Source: PLoS Pathog. 2012 Oct 4;8(10):e1002936. doi: 10.1371/journal.ppat.1002936 (PMC3464208; doi:10.1371/journal.ppat.1002936)
Supplement: Table S1 — C. neoformans strains used in this study. A subset of the in vitro revertants is listed. (PDF) [file ppat.1002936.s006.pdf]

Name	Genotype or phenotype	Background/other names	Isolation/generation	Reference
***C. neoformans* var. *neoformans* (serotype A)**				
KN99α	Wild type	H99		[1]
G	Wild type	ATCC 42347		[2]
C	*tao3*	ATCC 42343	Amoeba	[3]
D	*tao3*	ATCC 42344	Amoeba	[3]
E	*tao3*	ATCC 42345	Amoeba	[3]
FJW8	*cbk1::NAT*	KN99α	Targeted deletion of ORF	[4]
FJW9	*kic1::NAT*	KN99α	Targeted deletion of ORF	[4]
FJW10	*mob2::NAT*	KN99α	Targeted deletion of ORF	[4]
RM2	*tao3 T-DNA(NAT)*	KN99α	Insertional mutagenesis	[4]
DR3	*tao3*	KN99α	Insertional mutagenesis (unlinked)	[4]
AI217	*kic1 T-DNA(NEO) brh2::NAT*	KN99α	Insertional mutagenesis	This study
AI227	*tao3 T-DNA(NAT)*	KN99α	Insertional mutagenesis	This study
DM09	*mob2*	G	Amoeba	This study
DM09A	*mob2 MOB2-NEO*	DM09	Complementation	This study
AI255	*mob2 MOB2-NEO*	DM09	Complementation	This study
DM10	*tao3*	G	Amoeba	This study
AI222	*sog2::NAT*	KN99α	Targeted deletion of ORF	This study
AI235	*tao3::NAT*	KN99α	Targeted deletion of ORF	This study
AI235ya	*tao3::NAT* "Yellow"	AI235	Selected on FK506	This study
AI236	*tao3::NAT*	KN99α	Targeted deletion of ORF	This study
AI257	*tao3::NAT*, *MAT***a**	AI236 x KN99**a**	Progeny of cross	This study
AI194	*tao3::NAT*	G	Targeted deletion of ORF	This study
DM14	*tao3::NAT*	G	Targeted deletion of ORF	This study
AI210	Reconstituted *TAO3*^{BglII}	D	Reconstitution by gene replacement	This study
AI221	*tao3* revertant	D	Transformed and selected on FK506	This study
DM09-M3Ba	*mob2* revertant	DM09	Passage through mouse	This study
DM09-M3Bb	*mob2* revertant	DM09	Passage through mouse	This study
DM09-M3La	*mob2* revertant	DM09	Passage through mouse	This study
DM09-M3Lb	*mob2* revertant	DM09	Passage through mouse	This study
DM09-M4Ba	*mob2* revertant	DM09	Passage through mouse	This study
DM09-M4Bb	*mob2* revertant	DM09	Passage through mouse	This study
DM09-M4La	*mob2* revertant	DM09	Passage through mouse	This study
DM09-M4Lb	*mob2* revertant	DM09	Passage through mouse	This study
DM09-M5Ba	*mob2* revertant	DM09	Passage through mouse	This study
DM09-M5Bb	*mob2* revertant	DM09	Passage through mouse	This study
DM09-M5La	*mob2* revertant	DM09	Passage through mouse	This study
DM09-M5Lb	*mob2* revertant	DM09	Passage through mouse	This study

DM09-M7La	*mob2*	DM09	Passage through mouse	This study
DM09-M7Lb	*mob2*	DM09	Passage through mouse	This study
DM09-M10La	*mob2*	DM09	Passage through mouse	This study
DM09-M10Lb	*mob2*	DM09	Passage through mouse	This study
C. neoformans **var.** ***neoformans*** **(serotype D)**				
ATCC 24067	Wild type			[5]
ATCC 24067A	Passaged wild type isolate	ATCC 24067		[6]
F7	*tao3*	ATCC 24067A	UV or spontaneous	[7]
AI197	*tao3::NAT*	ATCC 24067A	Targeted deletion of ORF	This study
DM01	*tao3*	ATCC 24067A	Spontaneous (UV)	This study
DM02	*tao3*	ATCC 24067A	Spontaneous (UV)	This study
DM03	*sog2*	ATCC 24067A	Spontaneous (UV)	This study
DM04	*tao3*	ATCC 24067A	Spontaneous (UV)	This study
DM05	*tao3*	ATCC 24067A	Spontaneous (UV)	This study
AI209	*sog2 SOG2-NEO*	DM03	Complementation	This study
AI228	*cbk1*	ATCC 24067A	Spontaneous	This study
AI228 ura#3	*cbk1 ura5*	AI228	Selection of 5-FOA	This study
DM08	*cbk1 CBK1-NEO*	AI228	Complementation	This study
AI237	*mob2*	ATCC 24067A	Spontaneous (UV)	This study
DM11	*tao3*	ATCC 24067A	Amoeba	This study
DM12	*tao3*	ATCC 24067A	Amoeba	This study
DM13	*mob2*	ATCC 24067A	Amoeba	This study
AI242	$SOG2^{45aa}$	DM03	Selection on FK506	This study
AI243	*sog2* WT	DM03	Selection on FK506	This study
DM07	*SOG2*	DM03	Selection on FK506	This study
AI239	*TAO3*	F7	Selection of FK506	This study
AI240	$TAO3^{K \rightarrow L}$	F7	Selection of FK506	This study
AI241	*tao3* WT	F7	Selection of FK506	This study
DM06	$TAO3^{K \rightarrow Q}$	F7	Selection of FK506	This study
DM01a	*tao3* “Yellow”	DM01	Selection of FK506	This study
AI273	*tao3*	ATCC 24067A	Spontaneous	This study
AI274	*tao3*	ATCC 24067A	Spontaneous	This study
AI275	*tao3*	ATCC 24067A	Spontaneous	This study
AI276	*tao3*	ATCC 24067A	Spontaneous	This study
AI277	*kic1*	ATCC 24067A	Spontaneous	This study
AI278	*kic1*	ATCC 24067A	Spontaneous	This study
AI279	*kic1 KIC1-NAT*	AI277	Complementation	This study

References

1. Nielsen K, Cox GM, Wang P, Toffaletti DL, Perfect JR, et al. (2003) Sexual cycle of *Cryptococcus neoformans* var. *grubii* and virulence of congenic **a** and α isolates. Infect Immun 71: 4831-4841.
2. Neilson JB, Fromtling RA, Bulmer GS (1977) *Cryptococcus neoformans*: size range of infectious particles from aerosolized soil. Infect Immun 17: 634-638.
3. Neilson JB, Ivey MH, Bulmer GS (1978) *Cryptococcus neoformans*: pseudohyphal forms surviving culture with *Acanthamoeba polyphaga*. Infect Immun 20: 262-266.
4. Walton FJ, Heitman J, Idnurm A (2006) Conserved elements of the RAM signaling pathway establish cell polarity in the basidiomycete *Cryptococcus neoformans* in a divergent fashion from other fungi. Mol Biol Cell 17: 3768-3780.
5. Wilson DE, Bennett JE, Bailey JW (1968) Serologic grouping of *Cryptococcus neoformans*. Proc Soc Exp Biol Med 127: 820-823.
6. Franzot SP, Mukherjee J, Cherniak R, Chen LC, Hamdan JS, et al. (1998) Microevolution of a standard strain of *Cryptococcus neoformans* resulting in differences in virulence and other phenotypes. Infect Immun 66: 89-97.
7. Fries BC, Goldman DL, Cherniak R, Ju R, Casadevall A (1999) Phenotypic switching in *Cryptococcus neoformans* results in changes in cellular morphology and glucuronoxylomannan structure. Infect Immun 67: 6076-6083.
