# Supplementary material for: DNA Mutations Mediate Microevolution between Host-Adapted Forms of the Pathogenic Fungus Cryptococcus neoformans
Source: PLoS Pathog. 2012 Oct 4;8(10):e1002936. doi: 10.1371/journal.ppat.1002936 (PMC3464208; doi:10.1371/journal.ppat.1002936)
Supplement: Table S2 — Oligonucleotide primers used for amplification of DNA. (PDF) [file ppat.1002936.s007.pdf]

| Name | Sequence (5'-3') | Purpose |
|---|---|---|
| damp1 | CCGATACGATAAGCTAAG | *TAO3* gene replacement (var. *neoformans*) |
| damp2 | GCTTATGTGAGTCCTCCCACATCGTAAGTAGCGCCC | |
| damp3 | CTCGTTTCTACATCTCTTCGTGGTACGAGTGCTCTC | |
| damp4 | ATACGTGCCTTGTCAACC | |
| damp7 | TTGTGGGCAGCTTCACAG | *TAO3* gene replacement (var. *grubii*) |
| damp5 | GCTTATGTGAGTCCTCCCCGACTATCGCATTCCACC | |
| damp6 | CTCGTTTCTACATCTCTTCAAGACGAATGTTTCATC | |
| ALID0013 | TCGACGTAGATAGCTACAGGCG | |
| ALID0013 | TCGACGTAGATAGCTACAGGCG | Amplification of *TAO3* (var. *grubii*) |
| ALID0014 | CGTCGGTAACTTGAAATTCGGC | |
| ALID0060 | CCTTTATTTCTCAGCACC | |
| ALID0061 | AACTGCCACATGCGCTTG | |
| ALID0127 | GATATTGACTCAAGTGATGG | Amplification of *TAO3* (var. *neoformans*) |
| ALID0128 | ATCGAAATCCTCCTTGACAG | |
| ALID0129 | CGAGTCTTCACAGATTGCCC | |
| ALID0138 | AGGTGATAGAAGTCTGTC | |
| ALID0123 | GAAGTGAGATTGGCGATAAG | Amplification of *SOG2* |
| ALID0162 | TTTTGAAAAGCTCTCGACAG | |
| ALID0483 | TGTTGGAAGCTCAGCGTC | *SOG2* gene replacement |
| ALID0484 | GCTTATGTGAGTCCTCCCAACTCACTCTGAAGCTAG | |
| DM043 | CTCGTTTCTACATCTCTTTTATCTGAAGCGCTGGTC | |
| DM036 | CCATCTCGCTGATCTGTTAG | |
| ALID0111 | TCCCTGCCAGATACTGAC | Reconstitution of *TAO3* with *TAO3$^{BglII}$* construct |
| ALID0227 | TCAGGTGAGATCTTGCAATTCGAAGTAG | |
| ALID0228 | TTGCAAGATCTCACCTGATGACTCATAG | |
| ALID0061 | AACTGCCACATGCGCTTG | |
| ALID0977 | ATATGGCCTGAATTAAAGCC | Amplification of *CBK1* |
| ALID0978 | ATGTCGTAACTGGTCTAAGG | |
| DM062 | GCTTCCTCAACTCCATAG | Amplification of *MOB2* for probe and sequencing (var. *grubii*) |
| DM063 | CTTCTCACTCAATTCGGC | |
| ai006 | AAGAGATGTAGAAACGAG | Amplification of *NAT* cassette |
| ai290 | GGGAGGACTCACATAAGC | |
| ALID1675 | AGTGACAAGCACGATGTCGC | Amplification of *SOG2* for probe |
| ALID1676 | AGAGTTGCGTTGTGCAGGAG | |
| ALID1677 | GCAACAGAATATGCTGAACC | Amplification of *CBK1* for probe |
| ALID1678 | CGACAAGACACTCAAACATG | |
| ALID1685 | TAAAGTCGGTATACGGCAC | Amplification of *HYM1* for probe |
| ALID1687 | CATCATCATCTTCAGATTGG | |
| DM083 | GCTTCCTCAACTCGATCG | Amplification of *MOB2* for probe (var. *neoformans*) |
| DM084 | CTTCTCGCTCAACTCCGC | |
| ALID1681 | GCGTCTGGATCATCATGGAG | Amplification of *KIC1* for probe and sequencing (partial) |
| ALID1682 | ACGACCGGTTTGCTTGATGG | |
| ALID1679 | GATGGAAGAGATCGTCATCC | Amplification of *TAO3* for probe |
| ALID1680 | TTCGCGGATAAATCTATCGC | |

| | | |
|---|---|---|
| ai018<br>ai019 | `ATGGAAGAAGAAGGTACG`<br>`TTAGAAACACTTTCGGTG` | Amplification of *ACT1* for probe |
| ALID0375<br>ALID0376 | `TGCAAAGAGCGAAGTTGC`<br>`GATTGACAGCCAACAATC` | Amplification of *URA5* |
